# Loss of Function in *Mlo* Orthologs Reduces Susceptibility of Pepper and Tomato to Powdery Mildew Disease Caused by *Leveillula taurica*

**DOI:** 10.1371/journal.pone.0070723

**Published:** 2013-07-29

**Authors:** Zheng Zheng, Teruo Nonomura, Michela Appiano, Stefano Pavan, Yoshinori Matsuda, Hideyoshi Toyoda, Anne-Marie A. Wolters, Richard G. F. Visser, Yuling Bai

**Affiliations:** 1 Wageningen UR Plant Breeding, Wageningen University & Research Centre, Wageningen, The Netherlands; 2 Laboratory of Phytoprotection Science and Technology, Kinki University, Nara, Japan; 3 Department of Soil, Plant and Food Science, University of Bari, Bari, Italy; Virginia Tech, United States of America

## Abstract

Powdery mildew disease caused by *Leveillula taurica* is a serious fungal threat to greenhouse tomato and pepper production. In contrast to most powdery mildew species which are epiphytic, *L. taurica* is an endophytic fungus colonizing the mesophyll tissues of the leaf. In barley, *Arabidopsis*, tomato and pea, the correct functioning of specific homologues of the plant *Mlo* gene family has been found to be required for pathogenesis of epiphytic powdery mildew fungi. The aim of this study was to investigate the involvement of the *Mlo* genes in susceptibility to the endophytic fungus *L. taurica*. In tomato *(Solanum lycopersicum)*, a loss-of-function mutation in the *SlMlo1* gene results in resistance to powdery mildew disease caused by *Oidium neolycopersici*. When the tomato *Slmlo1* mutant was inoculated with *L. taurica* in this study, it proved to be less susceptible compared to the control, *S. lycopersicum* cv. Moneymaker. Further, overexpression of *SlMlo1* in the tomato *Slmlo1* mutant enhanced susceptibility to *L. taurica*. In pepper, the *CaMlo2* gene was isolated by applying a homology-based cloning approach. Compared to the previously identified *CaMlo1* gene, the *CaMlo2* gene is more similar to *SlMlo1* as shown by phylogenetic analysis, and the expression of *CaMlo2* is up-regulated at an earlier time point upon *L. taurica* infection. However, results of virus-induced gene silencing suggest that both *CaMlo1* and *CaMlo2* may be involved in the susceptibility of pepper to *L. taurica*. The fact that overexpression of *CaMlo2* restored the susceptibility of the tomato *Slmlo1* mutant to *O. neolycopersici* and increased its susceptibility to *L. taurica* confirmed the role of *CaMlo2* acting as a susceptibility factor to different powdery mildews, though the role of *CaMlo1* as a co-factor for susceptibility cannot be excluded.

## Introduction

Powdery mildews are conspicuous plant pathogens that comprise approximately 500 species and infect more than 1500 plant genera [1]. In Europe, the largest application of fungicides is for controlling powdery mildew diseases in agricultural and horticultural production [2]. The powdery mildew pathogen *Leveillula taurica* (Lév.) G. Arnaud is a serious fungal threat to pepper as well as tomato production. Heavy epidemics of powdery mildew disease could cause a significant yield loss up to 2 to 4 kg/m^2^ in greenhouse pepper production [3].

Besides repeated application of fungicides, powdery mildew diseases could be controlled by using resistant cultivars. In practice, breeding for resistance is mainly done by introgressing the resistance trait from wild species into the cultivated crop. In tomato, the *Lv* gene is the only resistance (R) gene identifed so far conferring resistance to *L. taurica*, which was found in a wild tomato accession of *Solanum chilense* and mapped on chromosome 12 [4]. In the *Capsicum* genus, several studies have been carried out to search for resistance to *L. taurica* [5], [6], [7], [8]. Five quantitative trait loci (QTLs) for resistance have been identified, with one of the QTLs, namely *Lt-9.1,* co-linearizing with the tomato *Lv* locus [9].

In principle, all resistance resources discovered from the wild accessions could be promising materials for isolation of potential R-genes and be used in resistance breeding. However, there are several weaknesses of using R-genes. First of all, the interspecific crossability barrier could restrict introgression of an R-gene from the resistant donor into cultivated species [10]. Even if the resistant donor can be easily crossed with the cultivated species, extensive backcrossing is required to remove undesirable traits. Secondly, transferring R-genes from one species into another does not guarantee that resistance conferred by the R-genes is retained in the receptor species in all cases. The function of an R-gene sometimes requires additional gene(s) in signaling pathways and/or metabolites [11], [12]. Thirdly, R-genes confer race-specific resistance, which could easily be overcome by new races of the pathogen in a short period.

Complementary to introgression of R-genes, a novel breeding strategy has been proposed, which is to disable plant susceptibility genes (S-genes) [13], [14]. In order to infect a plant species a pathogen should be able to suppress the plant's innate immunity by exploiting effector molecules to establish effector-triggered susceptibility. Plant genes, which are required for triggering susceptibility to pathogens and play a negative role in defense responses, are referred to as S-genes [13]. Impairment of the function of plant S-genes results in recessive resistance. One representive example is the *Mildew resistance Locus O* (*Mlo*) gene identified in barley. Loss-of-function mutants of the barley *Mlo* gene give resistance to powdery mildew (*Blumeria graminis* f. sp. *hordei*) and have been used in European barley cultivation for more than 30 years [15]. In addition to barley, mutations in *Mlo* orthologues result in recessively inherited powdery mildew resistance in *Arabidopsis* [16], tomato [17] and pea [18], [19]. In tomato, we have demonstrated that the recessive *ol-2* gene conferring resistance to the powdery mildew pathogen *Oidium neolycopersici* contains a 19-bp deletion in the coding region of the tomato *Mlo* ortholog, *SlMlo1* [17], [20]. So far, no natural powdery mildew isolates could break down the *mlo*-based resistance, which thus represents a broad-spectrum resistance and has become a successful example of using S-genes in crop protection for durable resistance.

*Mlo* susceptibility genes are part of a large family (the *Mlo* gene family) encoding a class of plant-specific proteins anchored in the plasma membrane by seven transmembrane domains [21], [22]. When functional MLO susceptibility proteins are lacking, powdery mildew fungi fail to enter their host. So far, *mlo*-based resistancerepresents a well-studied pre-penetration resistance conferred by cell wall appositions [16], [23], [24], [25]. To date, 15, 17, 7, 9 and 12 *Mlo* paralogs have been reported in the genomes of *Arabidopsis*, grape, wheat, maize and rice, respectively [16], [26], [27], [28], [29]. Of these paralogs, members of two phylogenetic clades, one specific for monocots and the other for dicots, play a role in susceptibility to powdery mildews. These members are characterized by the presence of a tetra-peptide (D/E-F-S/T-F) motif in the cytoplasmic region at the C-terminus of the protein [17], [26], [30], [31] and their response to powdery mildew infection at the early time points [17], [32], [33].

Besides *O. neolycopersici,* another powdery mildew fungus, *L. taurica,* can also infect tomato. *O. neolycopersici* develops its mycelium on the leaf surface and is distinguishable by the appearance of characteristic powder-like colonies on the adaxial side of the leaves. In contrast, *L. taurica* grows intercellularly inside the leaf. After a latency period of 3–4 weeks disease symptoms appear as chlorotic spots on the adaxial side of the leaves (see http://cals.arizona.edu/plp/plpext/diseases/vegetables/tomato/pm.htm) and fungal conidiophores appear as white powdery masses on the abaxial side of the leaf [34]. Based on the different colonization habit, *O. neolycopersici* is referred to as an epiphytic powdery mildew fungus, whereas *L. taurica* is referred to as endophytic. The aim of this study was to test, in pepper and tomato, whether *Mlo* homologs could have a role in the interaction with the endophytic powdery mildew species *L. taurica.*

When we started our work only one *Mlo* gene had been identified in pepper [30]. This *CaMlo1* gene was considered to be a mildew-effective *Mlo* ortholog of *SlMlo1* [18], [30]. We isolated a second *Mlo* gene in pepper, *CaMlo2*. During our study, the isolation and characterization of another allele of the same *CaMlo2* gene has been described by Kim and Hwang [35], accession number JN896629. Their results demonstrate that the *CaMllo2* gene is involved in cell death response as well as formation of reactive oxygen species (ROS). In addition, they showed that the *CaMlo2* gene was induced by the hemibiotrophic bacterial pathogen *Xanthomonas campestris* pv. *vesicatoria* (*Xcv*), the oomycete pathogen *Phytophthora capsici*, exogenous salicylic acid (SA), methyl viologen (MV), NaCl and drought stress treatment. Silencing of *CaMlo2* could induce resistance against *Xcv* in pepper, while overexpression of *CaMlo2* in *Arabidopsis* resulted in enhanced susceptibility to *Pseudomonas syringae* pv. *tomato* and *Hyaloperonospera arabidopsidis*.

In this study, we show that the tomato *Slmlo1* mutant which is fully resistant to *O. neolycopersici*, is also partially resistant to *L. taurica*. In pepper, the newly isolated *CaMlo2* gene was transcriptionally responsive to the penetration of *L. taurica* and silencing of either *CaMlo1* or *CaMlo2* reduced the susceptibility of pepper to *L. taurica*. Overexpression of *CaMlo2* could restore the susceptibility of the tomato *Slmlo* mutant *ol-2* to *O. neolycopersici*, and increase its susceptibility to *L. taurica*. We provide evidence indicating that at least one pepper *Mlo* homologue is involved in susceptibility to *L. taurica.*

## Results

### *L. taurica* is sensitive to mlo-based resistance in tomato

**Figure 1:**
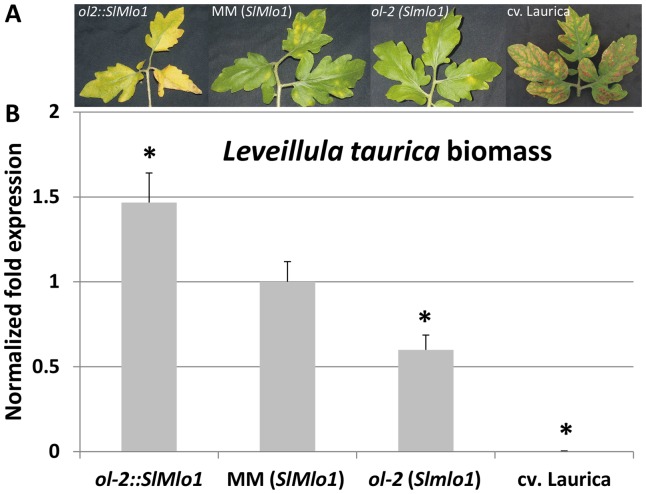
Results from artificial inoculation of *Leveillula taurica* on different tomato genotypes. **A.** From left to right: transformant of the breeding line *ol-2* in which the wild-type *SlMlo1* cDNA is overexpressed (*ol-2*::*SlMlo1*); cultivar Moneymaker (MM), homozygous for the wild-type *SlMlo1* allele; breeding line *ol-2,* homozygous for a *Slmlo1* loss-of-function allele; cultivar Laurica, carrying the *Lv* gene for hypersensitive response-based resistance. Yellow patches on the adaxial surface of *ol-2::SlMlo1*, MM and *ol-2* leaves are due to *L. taurica* colonization and correspond to abaxial fungal sporulation. Necrotic lesions on the Laurica genotype are the consequence of Lv-gene-mediated hypersensitive response and do not correspond to fungal growth on the abaxial side of the leaves. Pictures were taken four weeks after fungal inoculation. The experiment was carried out twice yielding similar results. **B.** Fungal DNA quantification by real-time PCR on the same genotypes described above. Amount of *L. taurica* DNA was normalized by the plant reference gene elongation factor (*SlEf*) with the ΔΔCt method. The relative pathogen biomass in MM plants is set as 1. In total, three leaves (4^th^, 5^th^ and 6^th^ leaf) per plant were pooled together, and three plants per genotype were assayed. Bars indicate the standard error of the mean for three biological replicates. Asterisks indicate significant difference with the control cultivar MM, performed by Student's t-test.

Previously, we reported that loss-of-function in the *SlMlo1* gene in tomato causes resistance against the powdery mildew pathogen *O. neolycopersici* [17]. In order to investigate whether *L. taurica* was principally amenable to *mlo*-based resistance, tomato lines carrying different alleles of the *SlMlo1* gene were challenged with *L. taurica*. Plants of the breeding line *ol-2* carrying the mutant allele of *SlMlo1* showed less symptoms and a significant decrease in fungal DNA amount compared to plants of the susceptible control Moneymaker (MM) (Figure 1). In contrast, plants of the *SlMlo1* over-expression line *ol2::SlMlo1* were more susceptible and showed a marked increase in fungal DNA amount compared to MM plants. It could be inferred that *SlMlo1* over-expression enhances the susceptibility to *L. taurica* in tomato and that loss-of-function in *SlMlo1* results in partial resistance against *L. taurica*. The resistant control cv. Laurica, harbouring the *Lv* gene, showed no fungal sporulation and a clear HR (hypersensitive response) phenotype upon *L. taurica* infection (Figure 1). No HR could be seen macroscopically in plants of the breeding line *ol-2*, indicating that the resistance in these plants is likely not conferred by the *Lv* gene (Figure 1). These data collectively indicate that in tomato *L. taurica* is sensitive to *mlo-*based resistance.

### Isolation of the *CaMlo2* gene in pepper

**Figure 2:**
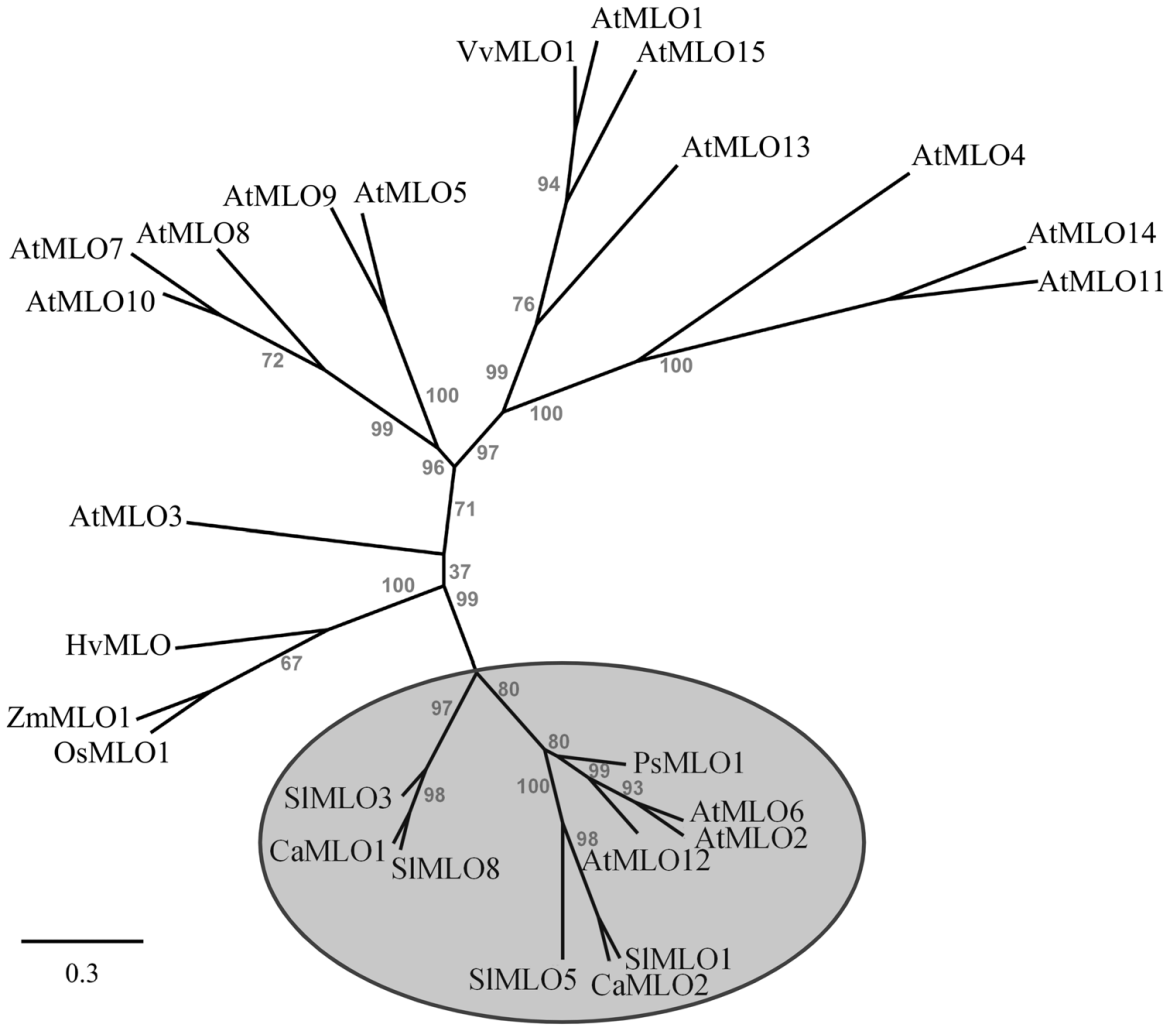
A phylogenetic tree of MLO proteins, showing that CaMLO1 and CaMLO2 are grouped together with AtMLO2, AtMLO6, AtMLO12 and SlMLO1, SlMLO3, SlMLO5 and SlMLO8 (grey-marked circle). This tree was constructed with Phylogeny.fr [44], using the radial view by TreeDyn. The unrooted radial tree comprises all 15 *Arabidopsis* MLO paralogs [28], tomato SlMLO1, SlMLO3, SlMLO5 and SlMLO8 as well as CaMLO1 and CaMLO2, in addition to pea PsMLO1, grape VvMLO1, barley HvMLO, rice OsMLO1 and maize ZmMLO1. The tree was established on the basis of an optimized multiple-sequence alignment. Numbers above nodes indicate bootstrap values (based on 100 replicates) that support the respective branch. The scale (lower left corner) indicates the number of amino acid exchanges per site.

**Figure 3:**
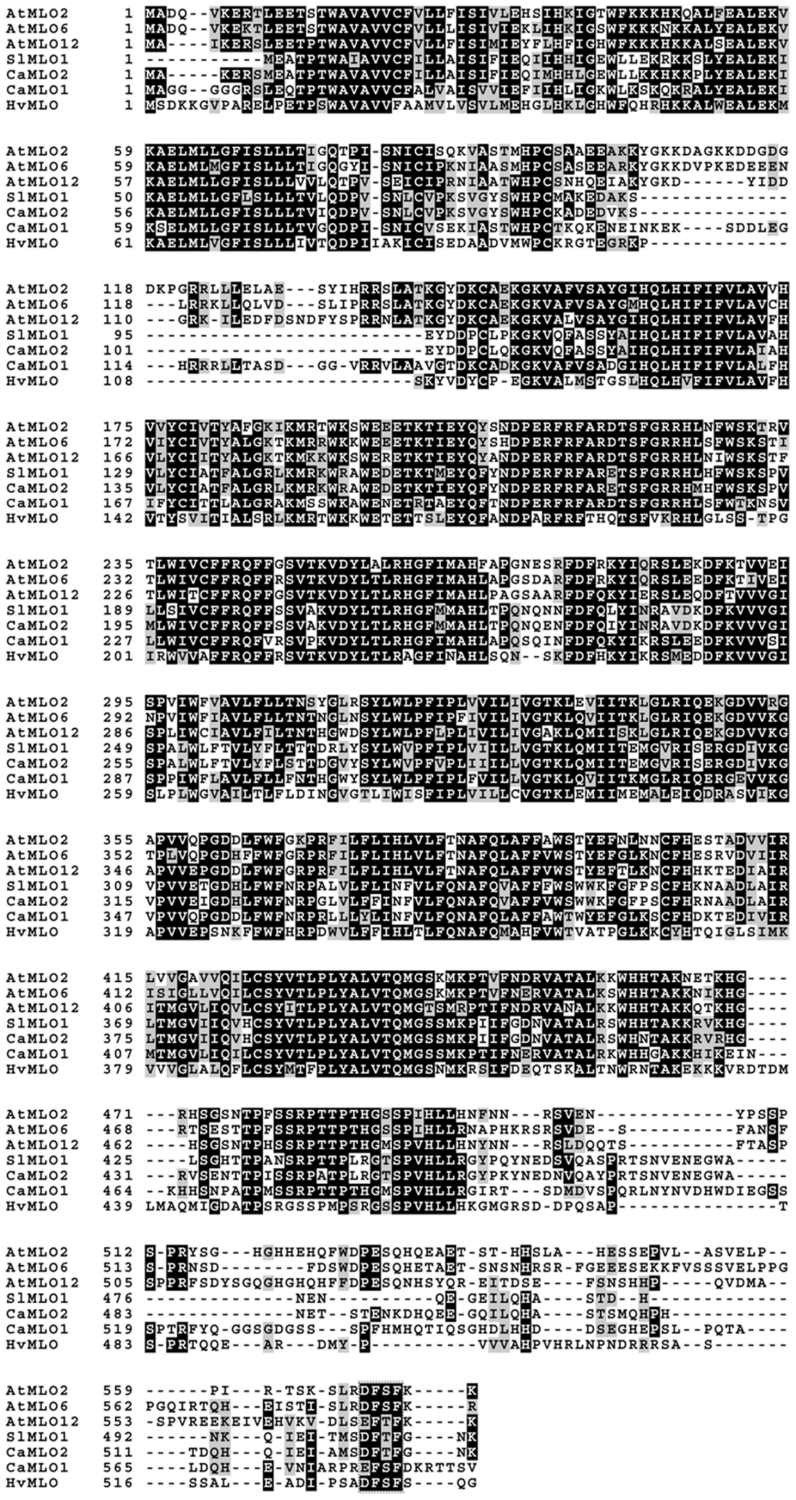
Multiple amino acid sequence alignment of pepper CaMLO1 and CaMLO2, barley HvMLO, tomato SlMLO1, as well as *Arabidopsis* AtMLO2, AtMLO6, and AtMLO12. The alignment was generated by CLUSTALW using default parameters. Grey boxes indicate similar amino acids and black boxes indicate identical amino acids. The C-terminal D/E-F-S/T-F tetra-peptide sequence, one of several motifs characteristic of barley *Mlo* orthologs [30] is outlined in grey. For this tetra-peptide sequence, CaMLO2 contains D-F-T-F, which is identical to SlMLO1.

The sensitivity of *L. taurica* to *mlo-*based resistance in tomato promotes the possibility to use loss-of-function mutations in pepper *Mlo* ortholog(s) for resistance to *L. taurica.* When we started our work, *CaMlo1* was the only identified *Mlo* homologue in pepper and considered as the ortholog of *SlMlo1* [18], [30]. By BLAST analysis, we inspected the publicly available pepper expressed sequence tag (EST) collections from the SOL Genomics Network (SGN) and identified one EST sequence (SGN-U202700) that is distinct from *CaMlo1*. The full-length cDNA sequence of this EST was obtained from cultivar Maor by Rapid Amplification of cDNA Ends (RACE), which was designated as *CaMlo2.* In the process of submitting this paper another report was published [35] in which the identification of another *CaMlo2* allele was described (GenBank accession number JN896629). Furthermore, in the newly released pepper transcriptome database [36] a *CaMlo2* sequence is present (accession number JW054099). The sequences of these three alleles were aligned (Figure S1A). The cDNA sequence of the Maor *CaMlo2* allele proved to be identical to sequence JW054099, while three SNPs were present compared with the JN896629 sequence. Protein sequences of all three *CaMlo2* alleles were identical. The *CaMlo2* cDNA sequences are clearly distinguishable from the *CaMlo1* cDNA sequences (Figure S1B). Phylogenetic analysis comprising all *Arabidopsis* as well as four tomato and the two pepper MLO protein sequences, and MLO protein sequences from additional plant species (Figure 2), revealed that the newly isolated CaMLO2 is closer to SlMLO1 than CaMLO1. Figure 3 shows the alignment of MLO proteins from *Arabidopsis*, tomato, pepper and barley. The C-terminal D/E-F-S/T-F tetra-peptide sequence, one of several motifs characteristic of barley *Mlo* orthologs [30], is outlined in grey dotted lines. For this tetra-peptide sequence CaMLO2 contains D-F-T-F, which is identical to SlMLO1, while CaMLO1 contains a different sequence (E-F-S-F). This supports the assumption that *CaMlo2* represents the pepper ortholog of *SlMlo1*.

### Accumulation of *CaMlo* transcripts upon *L. taurica* infection

**Figure 4:**
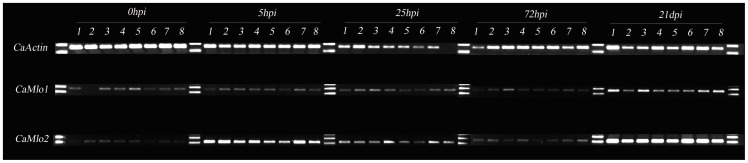
Expression of *CaMlo1* and *CaMlo2* in pepper upon *Leveillula taurica* infection measured by semi-quantitative RT-PCR at five time points: 0-hpi (hours post inoculation), 5 hpi, 25 hpi, 72 hpi and 21 dpi (days post inoculation). Leaf samples were collected from (in a left to right order): 1, susceptible cultivar A; 2, susceptible cultivar B; 3, susceptible cultivar Maor; 4, resistant doubled haploid HV-12; 5, Maor; 6, cultivar A; 7, HV-12; 8, cultivar B. Samples from different time points were separated by 1Kb marker (M). In total, three leaves per plant from each genotype were collected and leaves from each plant were pooled for RNA isolation. The analysis was carried out with a second primer set for *CaMlo1* and *CaMlo2*, yielding similar trends for expression.

Previously, we showed that the expression of *SlMlo1* is induced in tomato at early time points upon challenge with *O. neolycopersici* [17]. Data obtained by Piffanelli et al. [32] also demonstrated similar expression changes of the barley *Mlo* gene upon powdery mildew infection. In order to verify whether *CaMlo* genes are responsive to powdery mildew infection in pepper, we performed semi-quantitative RT-PCR and qRT-PCR in two independent experiments to study the expression of *CaMlo1* and *CaMlo2* in pepper plants which are susceptible to *L. taurica*. Results of the semi-quantitative RT-PCR showed an obvious induction of *CaMlo2* transcript at time points of 5 and 25 h post inoculation (hpi) and 21 days post inoculation (dpi) (Figure 4).

**Figure 5:**
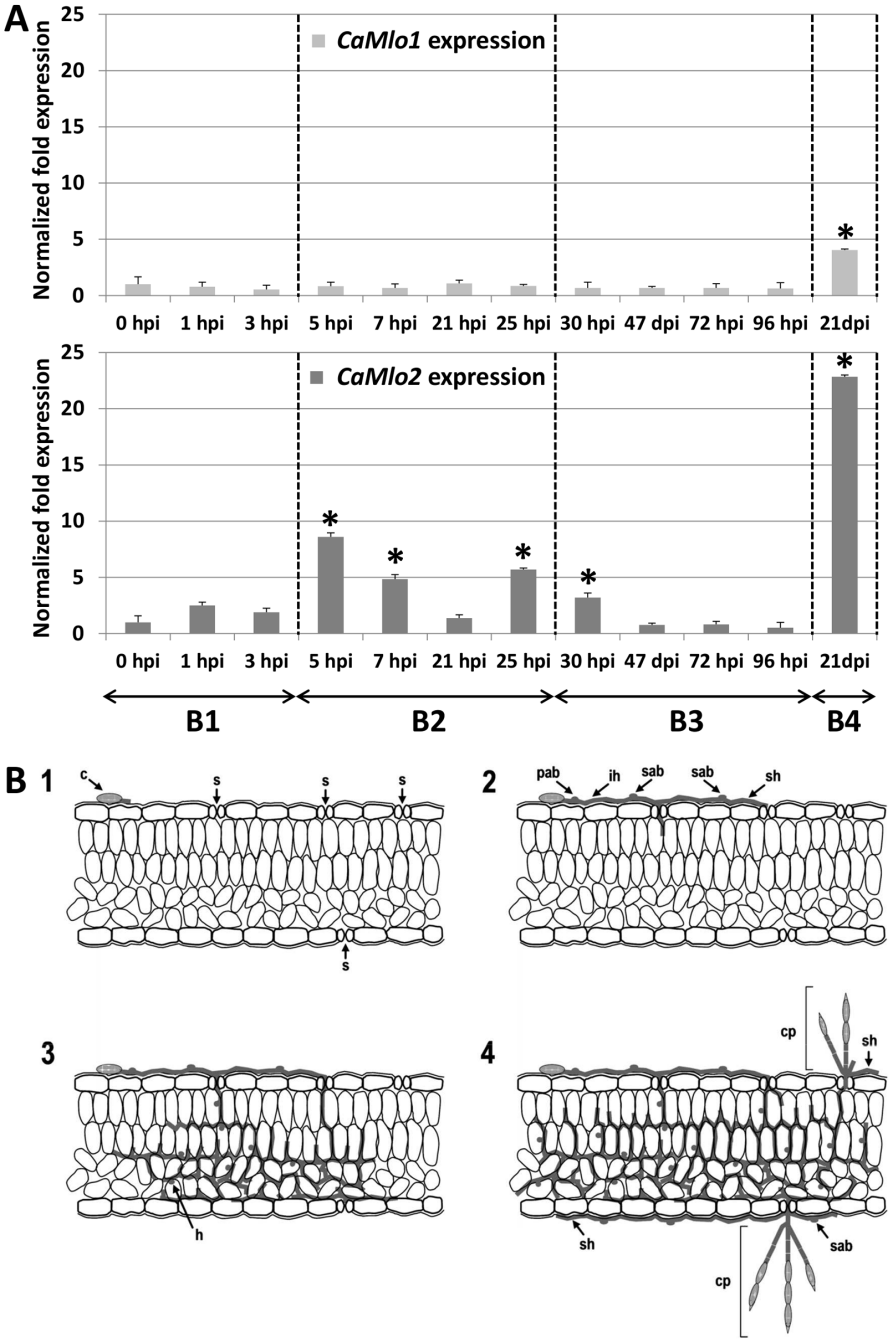
Accumulation of *CaMlo1* and *CaMlo2* transcripts during *Leveillula taurica* infection in pepper. **A.** Expression profile of *CaMlo* genes measured by real time qRT-PCR in pepper (cultivar A) leaves upon *Leveillula taurica* infection. Columns indicate transcript fold changes with respect to non-inoculated plants (0 hours after inoculation, hpi). Relative quantification was performed by using the ΔΔCt method and the reference gene *CaUEP*. Samples were taken from three whole pepper leaves per plant (the 3^rd^, 4^th^ and 5^th^ leaf) upon *L. taurica* infection at the following time points: 0 hpi, 1 hpi, 3 hpi, 5 hpi, 7 hpi, 21 hpi, 25 hpi, 30 hpi, 47 hpi, 72 hpi, 96 hpi and 21 days post inoculation (dpi). Results are based on three individual pepper plants per time point. Bars refer to standard errors of the biological replicates and asterisks refer to significant differences with respect to non-inoculated plants (0 hpi), inferred by mean comparisons with a Student's t-test. Time spans named as B1, B2, B3 and B4 refer to the fungal infection stages as described in the text and in panel B. Expression analyses were carried out with a different primer set for each *CaMlo* gene. **B.** Infection process of the endophytic powdery mildew *Leveillula taurica* on surface of pepper leaves. **1**, A conidium (c) germinates and a primary adhesion body (pab) is formed at the tip of the germ tube. **2**, Primary (infection) hyphae (ih) grow into the stomata (s) and secondary adhesion bodies (sab) are formed on the secondary hyphae (sh). **3**, The pathogen grows in the intercellular space and haustoria (h) are formed in mesophyll cells. **4**, Conidiophores (cp) are projected from the stomata three weeks after inoculation and superficial hyphae (sh) elongate on both sides of the leaves for a new round of infection.

In order to correlate the expression with the infection process, we consecutively tracked the infection progress of *L. taurica* on the pepper leaves with a digital microscope (Video S1) and performed qRT-PCR with more detailed time points (Figure 5A and Figure S2). Based on the infection process of *L. taurica*, we could cluster time points into four stages. The first stage (B1) corresponds to the period from 0 to 3 hpi. In this stage fungal spores landed on the leaf surface and started the germination, defined as fungal germination stage (Figure 5B–1). During this stage both *CaMlo* genes did not significantly change their expression level. The second stage (B2) includes four time points from 5 to 25-hpi. During this period, most spores formed a primary adhesion body (around 5 hpi), primary hyphae and secondary adhesion bodies (after 7 hpi). The adhesion bodies helped the fungus fix itself on the leaf surface and primary and secondary (branched) hyphae grew into the stomata (Figure 5B–2). This stage is defined as stomata penetration stage in this study. During this stage, the expression of *CaMlo2* was obviously induced at 5 hpi (the highest), 7 hpi and 25 hpi; in contrast, the expression of *CaMlo1* did not significantly vary. The third stage (B3), defined as fungal growing stage, includes four time points from 30 to 96 hpi. During this stage, intercellular growth (including haustorium formation in mesophyll cells) of *L. taurica* took place (Figure 5B–3) and the expression of *CaMlo* genes was quantified to be similar to the expression before fungal inoculation (with the exception of *CaMlo2* 30 hpi) (Figure 5A). The last time point is 21 dpi (stage B4), when *L. taurica* finished the whole life cycle and released the next generation spores (Figure 5B–4). Both *CaMlo1* and *CaMlo2* showed the highest expression at this time point. Taken together, these data show that the newly isolated *CaMlo2* gene is responsive to *L. taurica* at the stomata penetration stage, whereas *CaMlo1* expression is largely independent from the infection process, at least till four days after fungal inoculation. The gene expression levels were measured with two different primer pairs located in different regions of each gene and similar results were obtained.

### Silencing of *CaMlo* genes in pepper by VIGS

**Figure 6:**
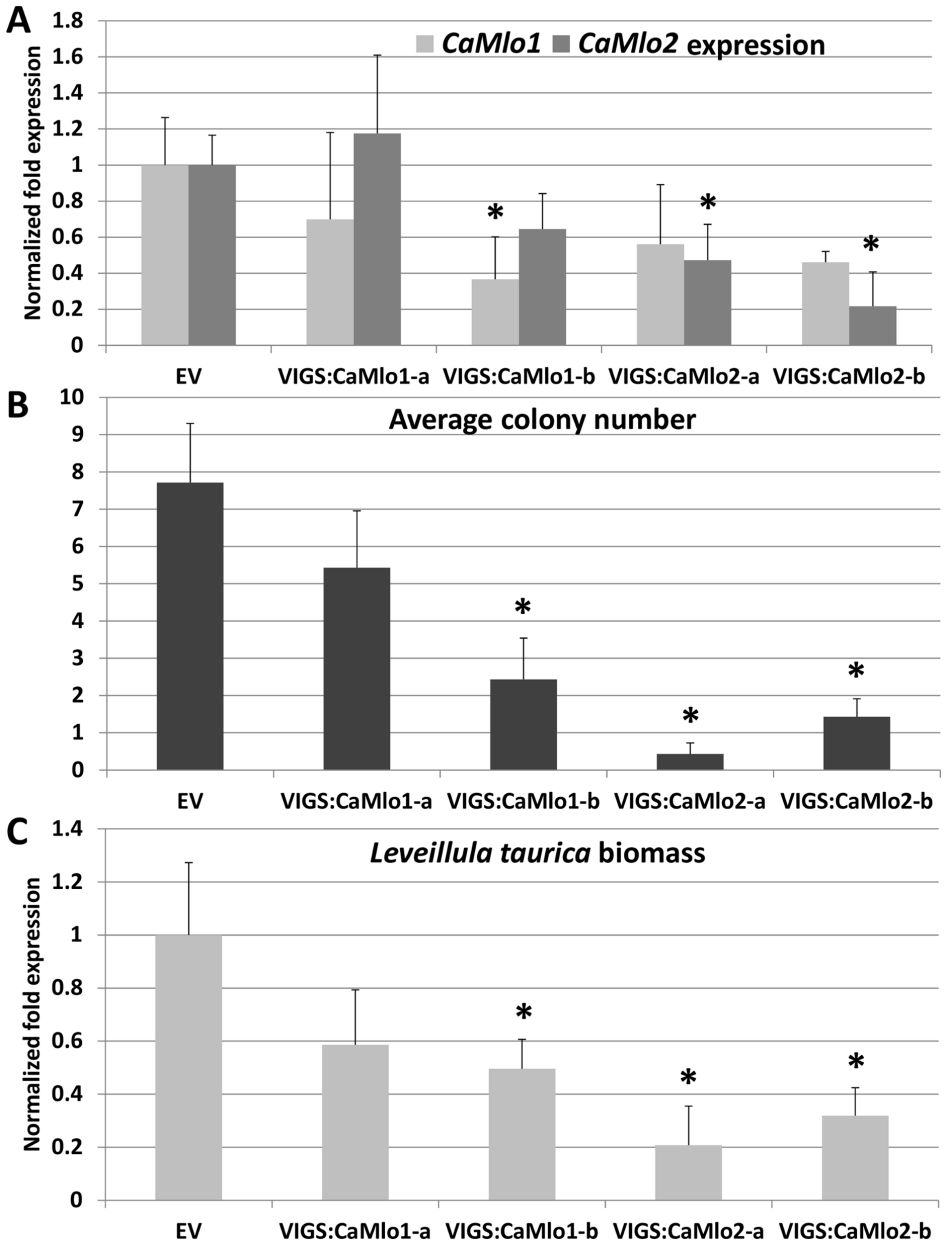
Effects of virus induced gene silencing (VIGS) for pepper *CaMlo1* and *CaMlo2*. **A.** Quantification of *CaMlo1* and *CaMlo2* gene expression in pepper plants (cv. A) subjected to VIGS. Two silencing constructs for each gene were developed (VIGS:CaMlo1-a and -b; VIGS:CaMlo2-a and -b). Columns refer to transcripts fold change with respect to plants inoculated with an empty TRV2 vector (EV plants). RNAs were isolated from pooled tissues of the 4^th^, 5^th^ and 6^th^ leaf for each plant. Relative quantification was performed by using the ΔΔCt method and the reference gene *CaActin*. Bars refer to standard errors of the mean of three biological replicates. Asterisks refer to significant differences with respect to expression levels of EV plants, inferred by means comparison with a Student's t-test. **B**. Quantification of *L. taurica* colonization levels on EV, VIGS:CaMlo1 (a and b) and VIGS:CaMlo2 (a and b) pepper plants (cv. A) by counting the average colony number on the 4^th^, 5^th^ and 6^th^ leaf for each plant. Bars refer to the standard error of the mean of seven plants for each treatment. Asterisks refer to significant differences with respect to EV plants, inferred by means comparison by Student's t-test. **C**. Quantification of *L. taurica* colonization levels on EV, VIGS:CaMlo1 (a and b) and VIGS:CaMlo2 (a and b) pepper plants (cv. A) by of fungal biomass by real time qPCR. DNAs were isolated from pooled 4^th^, 5^th^ and 6^th^ infected leaves per plant. In total, seven plants were tested for each VIGS vector. The ΔΔCt method with *CaActin* as the reference gene was used for normalization. Columns refer to relative quantification with respect to EV plants. Bars refer to standard errors referred to seven biological replicates. Asterisks refer to significant differences with respect to EV plants, inferred by means comparison by Student's t-test.

In order to evaluate the potential role of the *CaMlo* genes in conferring powdery mildew susceptibility in pepper, we performed functional analysis via VIGS. In total, four constructs were generated for *CaMlo1* and *CaMlo2* silencing (two constructs per gene), namely VIGS:CaMlo1-a, VIGS:CaMlo1-b, VIGS:CaMlo2-a and VIGS:CaMlo2-b. The sequences used for specific silencing of *CaMlo1* or *CaMlo2* are highlighted in Figure S1B. Plants infiltrated with the TRV2 empty vector (EV) were used as control. All EV-infiltrated plants showed symptoms when challenged with *L. taurica*, indicating that the TRV-based VIGS system does not influence the susceptibility of pepper to this fungus. Before fungal inoculation, *CaMlo* gene expression levels in plants infiltrated with the silencing constructs were assayed via qRT-PCR. For each construct except VIGS:CaMlo1-a, significant silencing of the target gene was observed (Figure 6A). However, silencing of *CaMlo1* in VIGS:CaMlo1-b plants was accompanied by a lower expression of *CaMlo2*, although this reduction was not significant. Similarly, a non-significant reduction of *CaMlo1* expression was observed in VIGS:CaMlo2 plants (Figure 6A). Compared with the EV control, plants silenced with each of the constructs except VIGS:CaMlo1-a showed a significant reduction of fungal colonies (visually measured) as well as fungal biomass (quantified by real-time PCR) (Figures 6B and C). In general, a stronger reduction was observed in VIGS:CaMlo2 plants compared to VIGS:CaMlo1 plants, suggesting that the newly isolated *CaMlo2* gene may play a major role in susceptibility to *L. taurica*. Two independent VIGS assays were performed and similar data were obtained. It is worth to note that, in the VIGS experiments, eight out of 10 VIGS:CaMlo2-a plants showed a remarkable decrease in size when compared with plants infiltrated with other VIGS constructs (Figure S3).

### Complementation of *SlMlo1* loss-of-function mutant by *CaMlo2*

**Figure 7:**
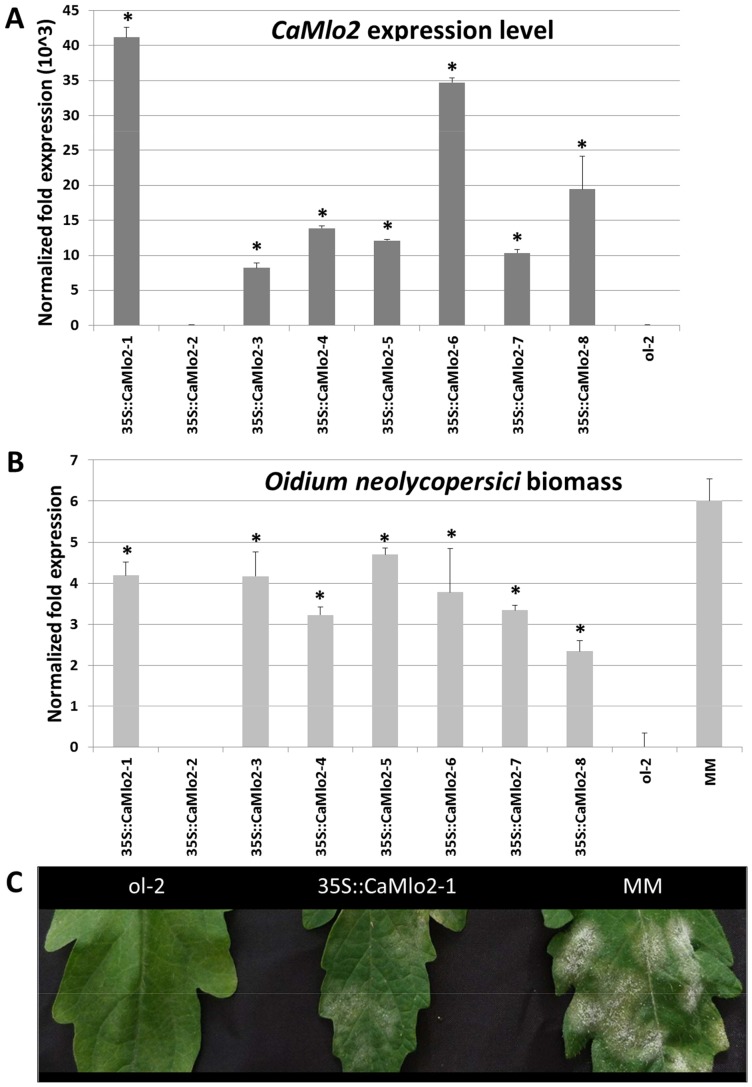
Results of the complementation of the *ol-2* tomato breeding line by *CaMlo2*. **A.** Quantification of the *CaMlo2* gene expression on a subset of T1 transgenic plants (35::CaMlo2-1 to -8). Columns refer to transcripts fold change with respect to the *ol-2* genotype. Bars refer to standard errors of the mean of three technical replicates for each T1 plant. Relative quantification was performed by using the ΔΔCt method and the reference gene *SlEf*. RNAs were isolated from tissues from the 5^th^ leaf for each plant. **B**. Quantification of *O. neolycopersici* performed by real-time qPCR on the same subset of T1 plants mentioned in **A**. Columns refer to the relative quantification of fungal biomass with respect to the fungal presence on *ol-2* plants. Bars refer to standard errors of the mean of three cuttings taken from each T1 tomato plant. The ΔΔCt method with *SlEf* as the reference gene was used for normalization. DNAs were isolated from the 2^nd^ or 3^rd^ leaf of each cutting. **C.** Transgenic overexpression of *CaMlo2* restores *Oidium neolycopersici* susceptibility in the *Slmlol* mutant. Leafs depicted show the outcome of the interaction 20 days after inoculation. ol-2  = ol-2 breeding line, homozygous for *SlMlo1* loss of function; 35S::CaMlo2-1  = a T1 plant obtained by overexpressing *CaMlo2* in the genetic background of the ol-2 line; MM  = the susceptible cv. MoneyMaker, homozygous for the wild-type *SlMlo1* allele.

To further confirm the role of *CaMlo2* in powdery mildew pathogenesis, *CaMlo2-*overexpressing transgenic plants (*35S::CaMlo2*) were obtained by transforming the tomato *Slmlo1* mutant (the *ol-2* mutant). First, we tested cuttings of the T1 transformants with *O. neolycopersici*. Results showed that overexpression of *CaMlo2* could restore susceptibility of *Slmlo1* mutant to *O. neolycopersici* (Figure 7), providing further evidence for the role of *CaMlo2* as a powdery mildew susceptibility factor. However, a fully restored susceptibility was not observed since the T1 transformants showed a significantly lower level of fungal biomass compared to cultivar Moneymaker (Figure 7), in addition to a 5-days delay of showing visible symptoms.

**Figure 8:**
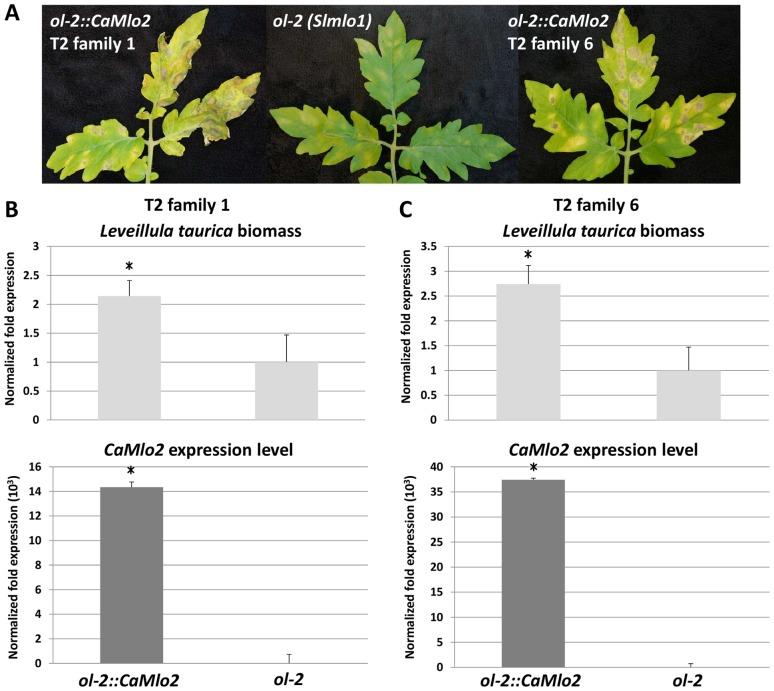
Assessment of *Leveillula taurica* susceptibility of *ol-2::CaMlo2* transformants. **A.** *L. taurica* disease symptoms on tomato mutant *ol-2* and two individual T2 plants of *CaMlo2*-overexpressing transformants of *ol-2*. **B.** and **C.** *L. taurica* biomass and *CaMlo2* expression level quantified by qRT-PCR of T2 plants expressing *CaMlo2* compared with *ol-2* plants for T2 family 1 (**B**) and T2 family 6 (**C**). Columns refer to relative quantification with respect to the *ol-2* plants. Bars refer to standard errors of the mean of 11 T2 plants of family 1, 12 T2 plants of family 6, and 10 *ol-2* plants. The ΔΔCt method with *SlEf* as the reference gene was used for normalization. DNA and RNA were isolated from pooled 2^nd^ and 3^rd^ leaves of each plant. Asterisks refer to significant differences with respect to *ol-2* plants, inferred by means comparison by Student's t-test.

T2 seeds were obtained from transformants 1 and 6, showing the highest *CaMlo2* expression level (Figure 7A). T2 plants, together with the *ol-2* mutant, were assayed for *L. taurica* susceptibility (Figure 8A). As the T2 families segregate for presence of the *35S::CaMlo2* gene insertion(s), the *CaMlo2* expression level of each T2 plant was measured by qRT-PCR. Figures 8B and 8C show that for both T2 families 1 and 6 the T2 plants showing expression of *CaMlo2* have a significantly higher *L. taurica* biomass than the *ol-2* plants. Therefore, we can conclude that *CaMlo2* plays a role in susceptibility to both the epiphytic powdery mildew *O. neolycopersici* and the endophytic powdery mildew *L. taurica.*

## Discussion

Previous studies documented that *Mlo* homologs are conserved across monocot and dicot plant species, and that mutations in certain *Mlo* homologs (e.g. barley *Mlo*, *Arabidopsis AtMlo2*, pea *PsMlo1* and tomato *SlMlo1)* result in resistance to epiphytic powdery mildew species [16], [26], [27], [28], [29]. However, this is the first study that shows that *Mlo* homologs can be also involved in susceptibility to an endophytic powdery mildew fungus.

In tomato, we found that *SlMlo1* overexpression increased susceptibility to *L. taurica* and in agreement with this the *Slmlo1* mutant was resistant to this fungus. However, compared with the full resistance to *O. neolycopersici*, the *Slmlo1* mutant was only partially resistant to *L. taurica*, In barley and pea, the mutation of a single *Mlo* gene suffices to confer full resistance against the adapted powdery mildews *B. graminis* f. sp. *hordei* and *E. pisi,* respectively. In contrast, in *Arabidopsis*, full penetration resistance to the adapted powdery mildew species *G. cichoracearum* and *G. orontii* is observed upon simultaneous loss-of-function of three *Mlo* homologues (*AtMlo2, AtMlo6* and *AtMlo12*). Here our data indicate that, for the plant species tomato, full resistance to *L. taurica* may require mutations in other *Mlo* genes in addition to *SlMlo1*. Recently, the tomato genome sequence has become available from The Tomato Genome Consortium (http://solgenomics.net/organism/ Solanum_lycopersicum/genome) [38], which provides us with the chance to perform a functional analysis of mildew-effective *SlMlo* paralogs to discover other tomato *Mlo* homologues which are potentially involved in the interaction between tomato and *L. taurica*.

In pepper, we isolated the *CaMlo2* gene and demonstrated that its expression was induced at the fungal penetration stage of *L. taurica*. Studies in tomato, barley and *Arabidopsis* have documented that mildew-effective *Mlo* gene(s) respond to fungal penetration at very early time points [16], [17], [18], [21], [32], [37]. In barley and tomato *Mlo* transcripts significantly increase 6 hours post inoculation (hpi) [17], [32]. Though both *CaMlo1* and *CaMlo2* transcripts accumulated during *L. taurica* infection, only *CaMlo2* transcripts showed a significant up-regulation at early time points (5 and 7-hpi), corresponding to the stage of first adhesion body formation. In contrast, *CaMlo1* was found to be significantly upregulated only at 21 dpi (Figures 4 and 5, and Video S1). Although we did not analyze *CaMlo1* and *CaMlo2* expression levels between 4 dpi and 21 dpi we do not expect major changes during this interval, based on our study of the infection process of *L. taurica* on pepper [40]. We observed that *L. taurica* hyphae penetrated pepper leaf tissue starting from 6 hours post inoculation (hpi). Primary hyphae penetrated leaf tissue within 24-hpi, whereas tips of secondary branched hyphae penetrated the leaf 30-48 hpi. Conidiophores emerged from stomata 14–16 dpi. Subsequently, mature conidia became the source of secondary infection around 21 dpi. In tomato inoculated with *O. neolycopersici SlMlo1* is induced only at early time points (6–36 hpi) [17]. Thus, it is expected that the *Mlo* susceptibility gene should be expressed at the time points of fungal penetration stage. Therefore, in pepper inoculated with *L. taurica* we measured expression at time points between 0 hpi and 4 dpi to monitor primary infection, and in addition at 21 dpi to monitor secondary infection.

Together with the evidence obtained in phylogenetic analysis, the transcriptional response to fungal infection suggests that *CaMlo2* is likely the ortholog of *SlMlo1*. *CaMlo1* shows highest homology to a different *SlMlo* gene that is not involved in tomato susceptibility to *O. neolycopersici* (unpublished results). If *CaMlo1* also responds at transcriptional level to *L. taurica*, it is likely to occur at later infection points during the intracellular fungal growing stage.

Surprisingly, knocking-down the expression of either *CaMlo1* or *CaMlo2* resulted in reduced susceptibility of pepper to *L. taurica,* suggesting that both homologues are involved in the susceptibility of pepper to this fungus. The alignment of *CaMlo1* and *CaMlo2* coding sequences (Figure S1B) shows there is no continuous stretch of 21 identical nucleotides present, which is the minimal requirement for efficient silencing. Still, we cannot completely rule-out co-silencing effects (Figure 6). Overexpression of *CaMlo2* restored the susceptibility of the tomato *Slmlo1* mutant to *O. neolycopersici*, and increased the susceptibility to *L. taurica*, thereby further indicating that *CaMlo2* is a pepper susceptibility gene towards different powdery mildew species (Figures 7 and 8). Also, it supports the assumption that *CaMlo2* is very likely the functional ortholog of *SlMlo1*. However, a more precise assessment of the biological role of *CaMlo1* and *CaMlo2* in the interaction with *L. taurica* needs future functional analyses. Currently, we are performing transformations to overexpress *CaMlo1* in the *Slmlo1* mutant and we are planning to retransform *CaMlo2*-expressing T2 plants with a *CaMlo1* overexpression construct. In case overexpression of *CaMlo1* increases susceptibility of the *Slmlo1* mutant to powdery mildew we plan to monitor the expression of *CaMlo1* during the intercellular fungal growing stage of *L. taurica*, i.e. between 4 and 21 dpi. With these materials and functional analysis, we hope to unravel the involvement of both *CaMlo1* and *CaMlo2* in the interaction between tomato or pepper and *L. taurica*. Meanwhile, we are carrying out an allele mining approach to search for natural mutations in *CaMlo* genes using publicly available germplasm.

*O. neolycopersici* is epiphytic and develops all structures except haustoria on the host surfaces. In contrast, *L. taurica* is endophytic because fungal hyphae grow intercellularly in the mesophyll. For complete resistance against *O. neolycopersici*, mutation of one *Mlo* homologue is sufficient [39]. In contrast, for complete resistance against *L. taurica*, our data suggest that in both tomato and pepper mutations in more than one *Mlo* gene may be required, as it is reported for *Arabidopsis*. A recent study showed that silencing of the *CaMlo2* gene could induce resistance against *Xanthomonas campestris* pv. *vesicatoria* in pepper [35]. Thus, the specificity or redundancy of plant MLO functions in susceptibility to pathogens in different pathosystems requires careful attention.

In general, resistances against plant diseases are achieved by introduction of R-genes into susceptible plant genotypes. With the recently proposed new S-genes breeding strategy, resistance could be achieved by impairment of susceptibility factors that evolved in the disease development [13], [14]. With increasing interest in the research topic on suppression of plant immunity, a considerable number of potential S-genes have been identified in *Arabidopsis*. However, it is largely unknown whether orthologs of *Arabidopsis S*-genes in crop species exist and are functional to corresponding crop pathogens. Taking *Mlo* as the target gene, this study shows that there are functional orthologs present in tomato and pepper, demonstrating the potential use of *S*-genes identified in *Arabidopsis* in breeding crops with durable resistance.

However, pleiotropic effects of the achieved resistance by disabling S-genes, as observed when silencing *CaMlo2*, should also be considered for practical applications. By comparing features of *Mlo*-like genes of several monocot species, the dicot *Arabidopsis* and the moss *Ceratodon purpureus*, it is assumed that the origin of the *Mlo* gene family can be tracked back at least to the early evolutionary stages of land plant development [28]. The ancient presence of plant *Ml*o gene families implies their vital function for plant development. In addition to being a negative regulator of plant defence, functions have been discovered for other members of the plant *Mlo* gene family. Barley and *Arabidopsis mlo* mutants showed some pleiotropic effect in older plants and under certain conditions, suggesting that mildew-effective MLOs may be involved in other biological functions as well [13]. However, the tomato *Slmlo1* mutant did not show an abnormal plant phenotype [17]. Thus, the functions of members of the *Mlo* gene family need to be further characterized.

## Materials and Methods

### Plant materials

Four tomato genotypes were used in this study: a breeding line *ol-2*, a homozygous T3 *ol-2* line (35S::*SlMlo1*), *Solanum lycopersicum* cv. Moneymaker (MM) and cv. Laurica. The *ol-2* line carries the mutated allele (a 19-bp deletion in the coding sequence) of *SlMlo1* and is resistant to *O. neolycopersici* [17]. Homozygous T3 *ol-2* line is the selfed progeny of a transgenic T2 plant over-expressing the *SlMlo1* gene under the 35S promoter (35S::*SlMlo1*) in the *ol-2* line and fully susceptible to *O. neolycopersici* [17]. MM carrying the natural *SlMlo1* allele was used as the susceptible control. Cultivar Laurica was used as the resistant control which carries the wild-type *SlMlo1* allele and the *Lv* gene derived from *S. chilense* LA1969.

Three pepper genotypes, cv. Maor and two anonymous cultivars A and B, were used which are all susceptible to *L. taurica*. The doubled-haploid line HV-12 (kindly provided by Dr.Alain Palloix, INRA, France) was used as the resistant control.

### *Leveillula taurica* disease assay

*L. taurica* was obtained in the greenhouse of a seeds company from The Netherlands and maintained on susceptible pepper plants in a climate chamber at Wageningen University, The Netherlands. The inoculation was performed on six weeks-old plants by a spray method. Conidiospores of *L. taurica* were washed off from heavily infected pepper leaves with water. Inoculum was adjusted to a final concentration of 2.5×10^4^ conidia/ml. The abaxial surface of plant leaves was spray-inoculated. After the inoculation, pepper plants were kept for 24 h in plastic cages with 100% relative humidity at 21°C and subsequently with a temperature of 21°C (day) / 19°C (night) and relative humidity of 65%. Inoculated tomato plants were kept in a greenhouse compartment without plastic cages, and thus without increasing the relative humidity to 100%.

For the analysis of *CaMlo1* and *CaMlo2* gene expression in pepper during infection with *L. taurica* two independent inoculations were performed. In the first one four genotypes (HV-12, Maor, cultivar A and cultivar B) were assayed, with two plants per genotype at five time points. In the second experiment three genotypes (Maor, cultivar A and cultivar B) were assayed, with three plants per genotype at 12 time points (including the five time points of the first experiment). For the disease assay of tomato transformants expressing *CaMlo2* in a *Slmlo1* mutant background (*ol-2*) T2 plants obtained from the self-pollination of T1 transformed plants 1 and 6 (Figure 7A) were used. For each of the two families15 plants were inoculated, together with 10 *ol-2* plants.

For scoring *L. taurica* symptoms, numbers of fungal colonies on the 4^th^, 5^th^ and 6^th^ true leaves were counted. Furthermore, Disease Index (DI) was used to score the sporulation of *L. taurica* on each infected leaf as described in [40]. This DI system was based on a scale of 0 to 5; 0 = no visible sporulation, 1 = restricted chlorotic spots on the adaxial leaf surface with weak or no sporulation on the corresponding abaxial leaf areas, 2 = several isolated sporulation spots on the abaxial leaf area, 3 = numerous sporulation spots covering up to 40–50% of the abaxial leaf area, 4 = numerous coalescent sporulation spots covering up to 75% of the abaxial leaf area, and 5 = the whole abaxial surface of the leaf, and also parts of the adaxial leaf surface covered with dense sporulation. The third, fourth and fifth true leaves were scored for each plant, for both tomato and pepper.

In addition, a real-time PCR method was used to quantify the fungal biomass. Internal Transcribed Spacer (ITS) sequences of *L. taurica* infecting pepper were retrieved from GenBank and specific primers LV-F (5′AGCCGACTAGGCTTGGTCTT3′) and LV-R (5′ GCGGGTATCCCTACCTGATT3′) were designed by using the online software: Primer3 plus (http://www.bioinformatics.nl/cgi-bin/primer3plus/primer3plus.cgi). The DNA was isolated from infected leaves. For tomato, *Ef* primers *SlEF*-F (5′GGAACTTGAGAAGGAGCCTAAG3′) and *SlEF*-R (5′CAACACCAACAGCAACAGTCT3′) [41] were used as reference to normalize the plant DNA proportion by ΔΔCt methods [42]. For pepper, *CaActin* primers *CaActin*-F (5′ATCCCTCCACCTCTTCACTCTC3′) and *CaActin*-R (5′GCCTTAACCATTCCTGTTCCATTATC3′) [43] were used as a reference.

### RNA/DNA isolation, RACE and sequence analysis

RNA and DNA were isolated by using the RNeasy Kit and DNeasy Kit (Qiagen, Germany) respectively, according to the manufacturer's recommendations. The Rapid Amplification of cDNA Ends (RACE) reaction was conducted using the GeneRacer^TM^ kit (Invitrogen). For obtaining the 5′ Untranslated Region (UTR) of the *CaMlo2* cDNA primer 5′R (5′TCTCTACAGTAATGAACTCGAGACAAA3′) was used together with the GeneRacer^TM^ 5′primer (5′CGACTGGAGCACGAGGACACTGA3′). For obtaining the 3′UTR primer 3′F (5′CGTGGGAATAAGTCCAGCAT3′) was used together with the GeneRacer^TM^ 3′primer (5′GCTGTCAACGATACGCTACGTAACG3′). Amplicons for sequencing were generated from 50 ng genomic DNA template. PCR amplifications were performed in 20 μl reactions using 1 u of Taq Polymerase, 1x reaction buffer, 200 nM dNTP and 250 nM of each primer. Standard cycling conditions were: 4 min initial denaturation at 94°C, followed by 35 cycles of 30 sec denaturation at 94°C, 30 sec at the appropriate annealing temperature, and 30–60 sec extension at 72°C. Reactions were finished by 7 min incubation at 72°C. PCR products were examined for quality on ethidium bromide or GelRed™-stained agarose gels. PCR products were directly sequenced on ABI377 or ABI3700 sequencers (Greenomics, Wageningen UR) using the dideoxy chain-termination method and ABI PRISM Reaction Kit. One of the amplification primers was used as sequencing primer. Sequences were aligned using CLUSTALW. Visualization of the alignment was performed using BOXSHADE 3.21 (http://www.ch.embnet.org/software/BOX_form.html). A phylogenetic tree was constructed using the “One Click” method of Phylogeny.fr [44] on http://www.phylogeny.fr/version2_cgi/index.cgi. The radial view was obtained by TreeDyn.

### Gene expression analysis

For gene expression analysis, the first experiment was conducted using a semiquantitative RT-PCR evaluating the expression of *CaMlo1* and *CaMlo2* at five time points after *L. taurica* inoculation: 0 hpi (hours post inoculation), 5 hpi, 25 hpi, 72 hpi and 21 dpi (days post inoculation). The 3^rd^, 4^th^ and 5^th^ leaves were collected from two individual plants of four different genotypes (HV12, Maor, cultivar A and cultivar B), and pooled per plant. cDNA was generated from 1 µg of total RNA using a iScript cDNA synthesis kit from Bio-Rad.

In a second independent inoculation experiment the 3^rd^, 4^th^ and 5^th^ leaves were collected from three individual plants of three different cultivars (cultivar A, cultivar B and Maor) at twelve time points: 0 hpi (hours post inoculation), 1 hpi, 3 hpi, 5 hpi, 7 hpi, 21 hpi, 25 hpi, 30 hpi, 47 hpi, 72 hpi, 96 hpi and 21 dpi (days post inoculation) after *L. taurica* inoculation. cDNAs was synthesized as described above. Quantitative real-time PCR was performed by using the SYBR^®^ Green dye on Bio-Rad iCycler iQ^®^ machine (Bio-Rad).

Primer sequences of *CaMlo1* and *CaMlo2* and the reference genes are shown in Table S1. Five pepper housekeeping genes were tested for expression stability in order to determine which could be used as a reference gene in qRT-PCR. These include *CaActin* [43], *Ubiquitin-conjugating protein* (*UBI-3*) [45], *Glyceraldehyde-3-phosphate dehydrogenase* (*GAPDH*) [45], *Elongation factor* (*Ef1α*) [46], and *Ubiquitin Extension Protein* (*UEP*) [46]. Gene expression stability was essayed with the BestKeeper program [47]. *CaActin*, *CaEF1α* and *CaUEP* proved to be most stable. These three reference genes were used in qRT-PCR analyses of two pepper cultivars A and B at twelve different time points after inoculation. Gene expression levels at different time points were normalized by ΔΔCt methods. All three reference genes yielded similar results for the two tested cultivars (Figure S2). Therefore, each of them could be used as a suitable reference gene in subsequent experiments.

### Virus Induced Gene silencing (VIGS) in pepper

The VIGS experiments were performed as described by Liu *et* *al*. [48]. Primer pairs (Table S2) were designed to amplify fragments suitable for specific silencing of *CaMlo1* or *CaMlo2* by using the online software Primer3 plus. Target fragments of *CaMlo1* were amplified and cloned into the pGEM®-T Easy vector (Promega). Positive plasmids were digested with *Eco*RI and target fragments were excised from an agarose gel. The pTRV2(pYL156) vector [37] was digested with *Eco*RI and dephosphorylation was performed by using Thermosensitive Alkaline Phosphatase (TSAP) (Promega). Target fragments of *CaMlo1* were ligated by T4 ligase into the linearized pTRV2 vector. Positive clones were confirmed by sequencing. Target fragments of *CaMlo2* were cloned into the Gateway-compatible vector pENTR D-TOPO (Invitrogen) and subsequently recombined into pTRV2-Gateway VIGS vector. The cloned sequences of *CaMlo1* and *CaMlo2* are highlighted in Figure S1B.

The pTRV2 vectors carrying the target gene fragment were transformed into *Agrobacterium* strain GV3101 by electroporation. A 100-mL culture of *Agrobacterium* containing the target vectors was grown overnight at 28°C in YEP (yeast extract/bactopeptone) medium with antibiotics (50 mg/mL kanamycin and 50 mg/mL rifampicin). The cells were resuspended into infiltration medium MMA (150 mM acetosyringone, 10 mM MgCl and 10 mM MES, pH 5.7) with OD600 = 2. Cultures were kept at room temperature for 1 to 6 hours before agroinfiltration. *Agrobacterium* strains containing the pTRV1 vector and pTRV2 were mixed at a 1:1 ratio and co-infiltrated into two weeks-old cotyledons of pepper. For each pTRV2 construct 10 pepper plants of cv. A were infiltrated. Infiltrated plants were grown at 22°C with a 16 h-light/8 h-dark photoperiod cycle. Three weeks after agroinfiltration, three of the ten plants were used for assessing the silencing effect by real-time PCR with the primers *CaMlo1-ge*-F and *CaMlo1-ge*-R for *CaMlo1* and primers *CaMlo2-ge*-F and *CaMlo2-ge*-R for *CaMlo2* (see above). The remaining seven plants were inoculated with *L. taurica*. Powdery mildew symptoms were observed three weeks after inoculation. Tested plants were scored for DI, and numbers of powdery mildew colonies were counted on the 4^th^, 5^th^ and 6^th^ leaves. After colony counting, these three leaves per plant were collected and pooled for DNA isolation and fungal quantification with qRT-PCR using primers LV-F and LV-R (see above).

### Consecutive digital micrographs by a dissecting microscope

Conidia were collected from conidiophores using a probe of an electrostatic spore collector and were transferred to particular sites of test leaves [49]. Conidial growth on host epidermal cells were photographed at 0.5–1 hour intervals after inoculation using a CCD-camera of a high-fidelity digital microscope KH-2700 (Hirox, Tokyo, Japan). Photographs were treated using an image processing software (Adobe Photoshop ver.5) (Adobe Systems, CA, USA), and 110 image-analyzed photographic data were input to a Windows live movie maker software (Microsoft, WA, USA) to present the animated data of the conidial development.

### Generation of transgenic ol-2 tomato plants expressing the *CaMlo2* gene

In order to amplify and clone the full length coding sequence of the pepper *CaMlo2* gene four different primer pairs were designed. An amplicon of the expected size was only obtained for *CaMlo2* with primers *CaMlo2*_4fw (5′caccATGGAGGCAACCCCTACGTGG3′) and *CaMlo2*_4rev (5′ CTATTTGTTTCCAAAAGTAAAATCTGACATT3′). This amplicon was cloned into Gateway-compatible vector pENTR D-TOPO (Invitrogen). Cloning reactions were performed in *E. coli* strain One Shot® TOP10 according to the manufacturer's instructions. Presence of the right fragment was assessed by colony PCR, restriction enzyme digestion and sequencing. Then, the amplicon was transferred by LR recombination to the binary vector pK7WG2 [50], which harbours a 35S Cauliflower Mosaic Virus (CaMV) promoter and the marker gene for kanamycin resistance *nptII*, following the manufacturer's instructions (Invitrogen). Recombinant plasmids were cloned into *E. coli* One Shot® TOP10. Bacterial colonies were screened for positive recombinant plasmids by colony PCR, restriction enzyme digestion and sequencing. Binary vectors containing the expected insert were subsequently transferred into the AGL1-*virG* strain of *Agrobacterium tumefaciens* [51] by electroporation. Transformation of the tomato *ol-2* mutant was performed according to [52]. The obtained T1 transformants and their corresponding T2 families were assessed for the expression of the target gene and the presence of the *nptII* marker gene by real-time qPCR, using the following primer pairs: *CaMlo2*_qPCR.2_fw (5′ TCACCTTGGAGAGTGGTTGTTG3′) with *CaMlo2*_qPCR.2_rev (5′GCGCAATTGC CAACACAAAG3′) and *nptII*_fw (5′ACTGGGCACAACAGACAATC3′) with *nptII*_rev (5′ TCGTCCTGCAGTTCATTCA G 3′). As housekeeping gene the elongation factor α was used (primers *SlEF*-F and *SlEF*-R).

### Disease assay with *O. neolycopersici*

Cuttings originating from 26 T1 transgenic plants (3 cuttings per T1 plant) were inoculated with the Wageningen isolate of *O. neolycopersici,* which has been maintained on cv. Moneymaker (MM) as described in [39]. Pathogen inoculation was carried out by using a inoculum suspension of 2.5×10^4^ conidia/ml. Three weeks after inoculation the disease severity was measured by real-time qPCR quantification of *O.neolycopesici* biomass (*On*). The infected leaves (the 2^nd^ or 3^rd^ leaf) sampled for each cutting. Plant and fungal DNAs were extracted by using the DNeasy DNA extraction kit (Qiagen, Germany). In total, 20 ng of DNA was used as template for amplification with the primer pair ON-F (5′-CGCCAAAGACCTAACCAAAA-3′) and ON-R (5′-AGCCAAGAGATCCGTTGTTG-3′), designed on *On*-specific ITS sequences (GenBank accession number EU047564). The *SlEF*-F *+ SlEF*-R primer pair was used as reference to normalize the plant DNA proportion by the ΔΔCt method.

Two T1 transformants (plants 1 and 6) showing the highest *CaMlo2* expression level and the most susceptible phenotype to *O. neolycopersici* (Figure 7), were selected to be self-pollinated. The resulting T2 families were used in a *L. taurica* assay as described in a previous paragraph.

## Supporting Information

Figure S1 **A.** Sequence alignment of cDNAs of different alleles of the *CaMlo2* gene. The alignment was generated by CLUSTAL 2.1 using default parameters. CaMlo2_cDNA_Maor indicates the cDNA sequence isolated from this study, JW054099 is a transcript from the pepper transcriptome database [36], and JN896629 indicates the cDNA identified in the study of Kim and Hwang [35]. Start and stop codons are indicated in green and red, respectively. Nucleotide differences are indicated in blue. **B.** Sequence alignment of coding sequences of *CaMlo1* cDNAs AY934528 and JW061356 and *CaMlo2* cDNAs JN896629 and JW054099. Identical nucleotides are boxed in black. Sequences used for VIGS experiments are highlighted in green and blue for CaMlo1 and yellow for *CaMlo2*. Primers used for qRT-PCR are indicated with blue arrows for *CaMlo1* and yellow arrows for *CaMlo2*.
(DOCX)

Figure S2 Expression profile of *CaMlo* genes measured by real time qRT-PCR in pepper leaves upon *Leveillula taurica* infection using three different reference genes. **A**, cultivar A. **B**, cultivar B. Columns indicate transcript fold changes with respect to non-inoculated plants (0 hours after inoculation (hpi)). Relative quantification was performed by using the ΔΔCt method and the reference genes *CaActin, CaEF1α* and *CaUEP*. Samples were taken from three whole pepper leaves per plant (the 3^rd^, 4^th^ and 5^th^ leaf) upon *L. taurica* infection at 0 hpi, 1 hpi, 3 hpi, 5 hpi, 7 hpi, 21 hpi, 25 hpi, 30 hpi, 47 hpi, 72 hpi, 96 hpi and 21 days post inoculation (dpi). Results are based on three individual pepper plants per time point. Bars refer to standard errors of the biological replicates and asterisks refer to significant differences with respect to non-inoculated plants, inferred by mean comparisons by Student's t-test.
(TIF)

Figure S3 Eight out of ten plants in which the *CaMlo2* gene is silenced with the VIGS construct CaMlo2-a (left) show an obvious decrease in size compared with plants in which *CaMlo1* is silenced (right).
(TIF)

Video S1 Infection progress of *Leveillula taurica* on pepper leaves. Germination of a primary conidium of *L. taurica* on the abaxial surface of a young pepper leaf (0 to 3 hours post inoculation, hpi), production of primary adhesion body followed by growing of primary hypha until the penetration of a stoma (3 to 24 hpi), and production of secondary adhesion bodies followed by emergence and growing of branched hyphae until penetration of stomata (24 to 48 hpi).
(WMV)

Table S1 Primers used for qRT-PCR gene expression analyses.
(DOCX)

Table S2 Primer pairs used to prepare VIGS constructs.
(DOCX)
