# Supplementary material for: Loss of Function in Mlo Orthologs Reduces Susceptibility of Pepper and Tomato to Powdery Mildew Disease Caused by Leveillula taurica
Source: PLoS One. 2013 Jul 29;8(7):e70723. doi: 10.1371/journal.pone.0070723 (PMC3726601; doi:10.1371/journal.pone.0070723)
Supplement: Figure S1 — A. Sequence alignment of cDNAs of different alleles of the CaMlo2 gene. The alignment was generated by CLUSTAL 2.1 using default parameters. CaMlo2_cDNA_Maor indicates the cDNA sequence isolated from this study, JW054099 is a transcript from the pepper transcriptome database [36], and JN896629 indicates the cDNA identified in the study of Kim and Hwang [35]. Start and stop codons are indicated in green and red, respectively. Nucleotide differences are indicated in blue. B. Sequence alignment of coding sequences of CaMlo1 cDNAs AY934528 and JW061356 and CaMlo2 cDNAs JN896629 and JW054099. Identical nucleotides are boxed in black. Sequences used for VIGS experiments are highlighted in green and blue for CaMlo1 and yellow for CaMlo2. Primers used for qRT-PCR are indicated with blue arrows for CaMlo1 and yellow arrows for CaMlo2. (DOCX) [file pone.0070723.s001.docx]

**Figure S1.** **A.** CLUSTAL 2.1 multiple sequence alignment of *CaMlo2* cDNA sequences. Start and stop codons are indicated in green and red, respectively. Nucleotide differences are indicated in blue.

CaMlo2_cDNA_Maor GTCTTTCTTATTGACATTTCCAGTCTTTCTCTTTCCATTAATACTTTTCTTCTTATCCCT 60

JW054099 GTCTTTCTTATTGACATTTCCAGTCTTTCTCTTTCCATTAATACTTTTCTTCTTATCCCT 60

JN896629 ------------------------------------------------------------

CaMlo2_cDNA_Maor GTTATTTATATATAGACAATCATAACTATACTCTTTTATCTTCAATTTCATAACTAACTA 120

JW054099 GTTATTTATATATAGACAATCATAACTATACTCTTTTATCTTCAATTTCATAACTAACTA 120

JN896629 ------------------------------------------------------------

CaMlo2_cDNA_Maor TACAATCATTGTGTAAAGGAATAGCTTATACTTTTATCTTCAATTGTGTAAAGAAAGAAA 180

JW054099 TACAATCATTGTGTAAAGGAATAGCTTATACTTTTATCTTCAATTGTGTAAAGAAAGAAA 180

JN896629 --CAATCATTGTGTAAAGGAATAGCTTATACTTTTATCTTCAATCGTGTAAAGAAAGAAA 58

start

CaMlo2_cDNA_Maor CAACAAAATCATTTTTGTTCTTCTCAAGGAAAATTCTCTATCAGAATTAATTGATGGCTA 240

JW054099 CAACAAAATCATTTTTGTTCTTCTCAAGGAAAATTCTCTATCAGAATTAATTGATGGCTA 240

JN896629 CAACAAAATCATTTTTGTTCTTCTCAAGGAAAATTCTCTATCAGAATTAATTGATGGCTA 118

CaMlo2_cDNA_Maor AAGAACGGTCGATGGAGGCAACCCCTACGTGGGCGGTTGCCGTGGTTTGCTTCATCTTGC 300

JW054099 AAGAACGGTCGATGGAGGCAACCCCTACGTGGGCGGTTGCCGTGGTTTGCTTCATCTTGC 300

JN896629 AAGAACGGTCGATGGAGGCAACCCCTACGTGGGCGGTTGCCGTGGTTTGCTTCATCTTGC 178

CaMlo2_cDNA_Maor TGGCTATTTCCATTTTTATTGAACAAATTATGCATCACCTTGGAGAGTGGTTGTTGAAAA 360

JW054099 TGGCTATTTCCATTTTTATTGAACAAATTATGCATCACCTTGGAGAGTGGTTGTTGAAAA 360

JN896629 TGGCTATTTCCATTTTTATTGAACAAATTATGCATCACCTTGGAGAGTGGTTGTTGAAAA 238

CaMlo2_cDNA_Maor AACACAAAAAGCCTCTATACGAAGCACTTGAAAAGATCAAAGCAGAGCTTATGTTGTTGG 420

JW054099 AACACAAAAAGCCTCTATACGAAGCACTTGAAAAGATCAAAGCAGAGCTTATGTTGTTGG 420

JN896629 AACACAAAAAGCCTCTATACGAAGCACTTGAAAAGATCAAAGCAGAGCTTATGTTGTTGG 298

CaMlo2_cDNA_Maor GATTCATATCATTGTTGTTGACAGTGATACAAGACCCAGTTTCTAACTTATGTGTCCCCA 480

JW054099 GATTCATATCATTGTTGTTGACAGTGATACAAGACCCAGTTTCTAACTTATGTGTCCCCA 480

JN896629 GATTCATATCATTGTTGTTGACAGTGATACAAGACCCAGTTTCTAACTTATGTGTCCCCA 358

CaMlo2_cDNA_Maor AAAGTGTTGGTTATTCCTGGCATCCTTGTAAGGCAGATGAAGATGTCAAGTCTGAGTATG 540

JW054099 AAAGTGTTGGTTATTCCTGGCATCCTTGTAAGGCAGATGAAGATGTCAAGTCTGAGTATG 540

JN896629 AAAGTGTTGGTTATTCCTGGCATCCTTGTAAGGCAGATGAAGATGTCAAGTCTGAGTATG 418

CaMlo2_cDNA_Maor ATGACCCTTGTTTACAAAAGGGAAAAGTTCAATTTGCATCTTCATATGCAATACACCAGC 600

JW054099 ATGACCCTTGTTTACAAAAGGGAAAAGTTCAATTTGCATCTTCATATGCAATACACCAGC 600

JN896629 ATGACCCTTGTTTACAAAAGGGAAAAGTTCAATTTGCATCTTCATATGCAATACACCAGC 478

CaMlo2_cDNA_Maor TCCATATCTTCATCTTTGTGTTGGCAATTGCGCATGTTTTGTACTGTATAGCAACTTTTG 660

JW054099 TCCATATCTTCATCTTTGTGTTGGCAATTGCGCATGTTTTGTACTGTATAGCAACTTTTG 660

JN896629 TCCATATCTTCATCTTTGTGTTGGCAATTGCGCATGTTTTGTACTGTATAGCAACTTTTG 538

CaMlo2_cDNA_Maor CTTTGGGCAGGTTAAAGATGAGAAAATGGAGGGCCTGGGAGGATGAAACAAAAACAATTG 720

JW054099 CTTTGGGCAGGTTAAAGATGAGAAAATGGAGGGCCTGGGAGGATGAAACAAAAACAATTG 720

JN896629 CTTTGGGGAGGTTAAAGATGAGAAAATGGAGGGCCTGGGAGGATGAAACAAAAACAATTG 598

CaMlo2_cDNA_Maor AGTACCAATTCTATAACGATCCTGAGAGGTTTAGGTTTGCAAGGGAGACCTCATTTGGAC 780

JW054099 AGTACCAATTCTATAACGATCCTGAGAGGTTTAGGTTTGCAAGGGAGACCTCATTTGGAC 780

JN896629 AGTACCAATTCTATAACGACCCTGAGAGGTTTAGGTTTGCAAGGGAGACCTCATTTGGAC 658

CaMlo2_cDNA_Maor GTAGGCATATGCATTTTTGGAGCAAGTCGCCGGTGATGCTCTGGATAGTTTGTTTCTTCA 840

JW054099 GTAGGCATATGCATTTTTGGAGCAAGTCGCCGGTGATGCTCTGGATAGTTTGTTTCTTCA 840

JN896629 GTAGGCATATGCATTTTTGGAGCAAGTCGCCGGTGATGCTCTGGATAGTTTGTTTCTTCA 718

CaMlo2_cDNA_Maor GGCAATTCTTTTCATCAGTAGCAAAAGTTGACTATTTAACCCTTAGACATGGGTTCATGA 900

JW054099 GGCAATTCTTTTCATCAGTAGCAAAAGTTGACTATTTAACCCTTAGACATGGGTTCATGA 900

JN896629 GGCAATTCTTTTCATCAGTAGCAAAAGTTGACTATTTAACCCTTAGACATGGGTTCATGA 778

CaMlo2_cDNA_Maor TGGCACATTTAACACCACAGAATCAAGAGAACTTTGATTTTCAAATATACATTAATAGAG 960

JW054099 TGGCACATTTAACACCACAGAATCAAGAGAACTTTGATTTTCAAATATACATTAATAGAG 960

JN896629 TGGCACATTTAACACCACAGAATCAAGAGAACTTTGATTTTCAAATATACATTAATAGAG 838

CaMlo2_cDNA_Maor CAGTTGACAAAGATTTCAAAGTTGTCGTGGGAATAAGTCCAGCATTATGGCTCTTCACGG 1020

JW054099 CAGTTGACAAAGATTTCAAAGTTGTCGTGGGAATAAGTCCAGCATTATGGCTCTTCACGG 1020

JN896629 CAGTTGACAAAGATTTCAAAGTTGTCGTGGGAATAAGTCCAGCATTATGGCTCTTCACGG 898

CaMlo2_cDNA_Maor TATTATATTTTCTATCCACCACCGATGGAGTTTACTCGTATCTTTGGGTTCCATTTGTCC 1080

JW054099 TATTATATTTTCTATCCACCACCGATGGAGTTTACTCGTATCTTTGGGTTCCATTTGTCC 1080

JN896629 TATTATATTTTCTATCCACCACCGATGGAGTTTACTCGTATCTTTGGGTTCCATTTGTCC 958

CaMlo2_cDNA_Maor CACTCATTATAATATTGTTGGTTGGGACAAAACTTCAAATGATCATAACAGAAATGGGGG 1140

JW054099 CACTCATTATAATATTGTTGGTTGGGACAAAACTTCAAATGATCATAACAGAAATGGGGG 1140

JN896629 CACTCATTATAATATTGTTGGTTGGGACAAAACTTCAAATGATCATAACAGAAATGGGGG 1018

CaMlo2_cDNA_Maor TTAGAATTTCAGAAAGGGGAGACATAGTGAAAGGTGTACCAGTGGTGGAGATCGGTGACC 1200

JW054099 TTAGAATTTCAGAAAGGGGAGACATAGTGAAAGGTGTACCAGTGGTGGAGATCGGTGACC 1200

JN896629 TTAGAATTTCAGAAAGGGGAGACATAGTGAAAGGTGTACCAGTGGTGGAGATCGGTGACC 1078

CaMlo2_cDNA_Maor ATCTTTTCTGGTTTAATCGCCCTGGCCTTGTGCTTTTCTTCATTAACTTTGTCCTCTTTC 1260

JW054099 ATCTTTTCTGGTTTAATCGCCCTGGCCTTGTGCTTTTCTTCATTAACTTTGTCCTCTTTC 1260

JN896629 ATCTTTTCTGGTTTAATCGCCCTGGCCTTGTGCTTTTCTTCATTAACTTTGTCCTCTTTC 1138

CaMlo2_cDNA_Maor AGAATGCGTTTCAAGTTGCTTTCTTTGTTTGGAGTTGGTGGAAATTTGGTTTTCCATCCT 1320

JW054099 AGAATGCGTTTCAAGTTGCTTTCTTTGTTTGGAGTTGGTGGAAATTTGGTTTTCCATCCT 1320

JN896629 AGAATGCGTTTCAAGTTGCTTTCTTTGTTTGGAGTTGGTGGAAATTTGGTTTTCCATCCT 1198

CaMlo2_cDNA_Maor GCTTTCATAGAAATGCTGCAGACCTAGCCATTAGGCTAACCATGGGAGTAATCATACAAG 1380

JW054099 GCTTTCATAGAAATGCTGCAGACCTAGCCATTAGGCTAACCATGGGAGTAATCATACAAG 1380

JN896629 GCTTTCATAGAAATGCTGCAGACCTAGCCATTAGGCTAACCATGGGAGTAATCATACAAG 1258

CaMlo2_cDNA_Maor TCCATTGCAGCTATGTAACTCTCCCTCTATATGCCTTAGTTACTCAGATGGGTTCATCAA 1440

JW054099 TCCATTGCAGCTATGTAACTCTCCCTCTATATGCCTTAGTTACTCAGATGGGTTCATCAA 1440

JN896629 TCCATTGCAGCTATGTAACTCTCCCTCTATATGCCTTAGTTACTCAGATGGGTTCATCAA 1318

CaMlo2_cDNA_Maor TGAAGCCTATCATCTTTGGTGATAATGTGGCAACAGCTCTTAGAAGCTGGCACAATACAG 1500

JW054099 TGAAGCCTATCATCTTTGGTGATAATGTGGCAACAGCTCTTAGAAGCTGGCACAATACAG 1500

JN896629 TGAAGCCTATCATCTTTGGTGATAATGTGGCAACAGCTCTTAGAAGCTGGCACAATACAG 1378

CaMlo2_cDNA_Maor CGAAAAAGCGGGTGAGACATGGGCGGGTATCAGAAAACACCACTCCGATATCTAGCAGAC 1560

JW054099 CGAAAAAGCGGGTGAGACATGGGCGGGTATCAGAAAACACCACTCCGATATCTAGCAGAC 1560

JN896629 CGAAAAAGCGGGTGAGACATGGGCGGGTATCAGAAAACACCACTCCGATATCTAGCAGAC 1438

CaMlo2_cDNA_Maor CGGCCACACCATTGCGTGGTACCTCCCCAGTTCACTTGCTACGTGGCTACCCAAAATATA 1620

JW054099 CGGCCACACCATTGCGTGGTACCTCCCCAGTTCACTTGCTACGTGGCTACCCAAAATATA 1620

JN896629 CGGCCACACCATTGCGTGGTACCTCCCCAGTTCACTTGCTACGTGGCTACCCAAAATATA 1498

CaMlo2_cDNA_Maor ACGAGGACAATGTTCAAGCATATCCTCGAACATCGAATGTAGAAAATGAAGGCTGGGCTA 1680

JW054099 ACGAGGACAATGTTCAAGCATATCCTCGAACATCGAATGTAGAAAATGAAGGCTGGGCTA 1680

JN896629 ACGAGGACAATGTTCAAGCATATCCTCGAACATCGAATGTAGAAAATGAAGGCTGGGCTA 1558

CaMlo2_cDNA_Maor ATGAAACATCCACTGAGAATAAAGATCATCAGGAGGAGGGACAAATCCTGCAGCATGCCT 1740

JW054099 ATGAAACATCCACTGAGAATAAAGATCATCAGGAGGAGGGACAAATCCTGCAGCATGCCT 1740

JN896629 ATGAAACATCCACTGAGAATAAAGATCATCAGGAGGAGGGACAAATCCTGCAGCATGCCT 1618

CaMlo2_cDNA_Maor CCACTTCTATGCAACATCCGCATACTGATCAACATCAAATTGAGATTGCAATGTCAGATT 1800

JW054099 CCACTTCTATGCAACATCCGCATACTGATCAACATCAAATTGAGATTGCAATGTCAGATT 1800

JN896629 CCACTTCTATGCAACATCCGCATACTGATCAACATCAAATTGAGATTGCAATGTCAGATT 1678

stop

CaMlo2_cDNA_Maor TTACTTTTGGAAACAAATAG---------------------------------------- 1820

JW054099 TTACTTTTGGAAACAAATAGATCAATTGATCATCTAGTCTCCATATGTTAAAACTTCCAT 1860

JN896629 TTACTTTTGGAAACAAATAGATCAATTGATCATCTAGTCTCCATATGTTAAAACTTCCAT 1738

CaMlo2_cDNA_Maor ------------------------------------------------------------

JW054099 CTTCATTGTTTTCTTTGTCTCGAGTTCATTACTGTAGAGAATACCGTCTTCATTGTTTCC 1920

JN896629 CTTCATTGTTTTCTTTGTCTCGAGTTCATTACTGTAGAGAATACCGTCTTCATTGTTTCC 1798

CaMlo2_cDNA_Maor ------------------------------------------------------------

JW054099 TTTGTTTCGAGTTCTTTACTGTAGAGAATATATGTATATGTGTG---------------- 1964

JN896629 TTTGTTTCGAGTTCTTTACTGTAGAGAATATATGTATATGTGTGTGTATATATATATATA 1858

CaMlo2_cDNA_Maor ----------

JW054099 ----------

JN896629 TATTTATATA 1868

**Figure S1. B.** CLUSTALW multiple sequence alignment of coding sequences (CDS) of *CaMlo1* cDNAs AY934528 and JW061356 and *CaMlo2* cDNAs JN896629 and JW054099. Identical nucleotides are boxed in black. Sequences used for VIGS experiments are highlighted in green and blue for *CaMlo1* and yellow for *CaMlo2*. Primers used for qRT-PCR are indicated with blue arrows for *CaMlo1* and yellow arrows for *CaMlo2*.

JN896629_CDS ---------------------------ATGGAGGCAACCCCTACGTGGGCGGTTGCCGTG 33

JW054099_CDS ---------------------------ATGGAGGCAACCCCTACGTGGGCGGTTGCCGTG 33

AY934528_CDS ATGGCGGGAGGAGGGGGAGGAAGATCGTTGGAGCAAACGCCGACGTGGGCGGTTGCCGTA 60

JW061356_CDS ATGGCGGGAGGAGGGGGAGGAAGATCGTTGGAGCAAACGCCGACGTGGGCGGTTGCCGTA 60

***** *** ** *****************

JN896629_CDS GTTTGCTTCATCTTGCTGGCTATTTCCATTTTTATTGAACAAATTATGCATCACCTTGGA 93

JW054099_CDS GTTTGCTTCATCTTGCTGGCTATTTCCATTTTTATTGAACAAATTATGCATCACCTTGGA 93

AY934528_CDS GTTTGTTTTGCGTTGGTTGCTATTTCTGTCGTAATAGAGTTCATCATCCATCTTATTGGC 120

JW061356_CDS GTTTGTTTTGCGTTGGTTGCTATTTCTGTCGTAATAGAGTTCATCATCCATCTTATTGGC 120

***** ** *** * ******** * * ** ** ** ** **** ****

JN896629_CDS GAGTGGTTGTTGAAAAAACACAAAAAGCCTCTATACGAAGCACTTGAAAAGATCAAAGCA 153

JW054099_CDS GAGTGGTTGTTGAAAAAACACAAAAAGCCTCTATACGAAGCACTTGAAAAGATCAAAGCA 153

AY934528_CDS AAGTGGTTGAAGTCCAAACAAAAAAGAGCATTATATGAAGCACTTGAGAAGATAAAATCA 180

JW061356_CDS AAGTGGTTGAAGTCCAAACAAAAAAGAGCATTATATGAAGCACTTGAGAAGATAAAATCA 180

******** * ***** **** * **** *********** ***** *** **

JN896629_CDS GAGCTTATGTTGTTGGGATTCATATCATTGTTGTTGACAGTGATACAAGACCCAGTTTCT 213

JW054099_CDS GAGCTTATGTTGTTGGGATTCATATCATTGTTGTTGACAGTGATACAAGACCCAGTTTCT 213

AY934528_CDS GAATTAATGTTGTTGGGATTTATATCCCTACTACTAACAGTAGGGCAAGATCCAATTTCA 240

JW061356_CDS GAATTAATGTTGTTGGGATTTATATCCCTACTACTAACAGTAGGGCAAGATCCAATTTCA 240

** * ************** ***** * * * ***** ***** *** ****

JN896629_CDS AACTTATGTGTCCCCAAAAGTGTTGGTTATTCCTGGCATCCTTGTA---------AGGCA 264

JW054099_CDS AACTTATGTGTCCCCAAAAGTGTTGGTTATTCCTGGCATCCTTGTA---------AGGCA 264

AY934528_CDS AATATTTGTGTATCTGAAAAAATTGCTAGTACATGGCATCCATGTACTAAGCAAAAAGAA 300

JW061356_CDS AATATTTGTGTATCTGAAAAAATTGCTAGTACATGGCATCCATGTACTAAGCAAAAAGAA 300

** * ***** * *** *** * * * ******** **** * * *

JN896629_CDS GATGAA------------------GATG----------TCA------------------- 277

JW054099_CDS GATGAA------------------GATG----------TCA------------------- 277

AY934528_CDS AATGAAATAAATAAAGAAAAGTCCGATGACTTAGAGGGTCATCGCCGGCGACTACTTACG 360

JW061356_CDS AATGAAATAAATAAAGAAAAGTCCGATGACTTAGAGGGTCATCGCCGGCGACTACTTACG 360

***** **** ***

JN896629_CDS --------------AGTCTG---AGTAT-GATGACCCTTG---TTTACAAA--------- 307

JW054099_CDS --------------AGTCTG---AGTAT-GATGACCCTTG---TTTACAAA--------- 307

AY934528_CDS GCTTCTGATGGCGGAGTCCGGCGAGTTTTGGCGGCTGTTGGAACCGACAAATGTGCGGAT 420

JW061356_CDS GCTTCCGATGGCGGAGTCCGGCGAGTTTTGGCGGCTGTTGGAACCGACAAATGTGCGGAT 420

**** * *** * * * * *** *****

JN896629_CDS -AGGGAAAAGTTCAATTTGCATCTTCATATGCAATACACCAGCTCCATATCTTCATCTTT 366

JW054099_CDS -AGGGAAAAGTTCAATTTGCATCTTCATATGCAATACACCAGCTCCATATCTTCATCTTT 366

AY934528_CDS AAGGGAAAAGTAGCATTTGTGTCTGCAGATGGAATTCATCAATTACATATTTTTATTTTT 480

JW061356_CDS AAGGGAAAAGTAGCATTTGTGTCTGCAGATGGAATTCATCAATTACATATTTTTATTTTT 480

********** ***** *** ** *** *** ** ** * ***** ** ** ***

JN896629_CDS GTGTTGGCAATTGCGCATGTTTTGTACTGTATAGCAACTTTTGCTTTGGGGAGGTTAAAG 426

JW054099_CDS GTGTTGGCAATTGCGCATGTTTTGTACTGTATAGCAACTTTTGCTTTGGGCAGGTTAAAG 426

AY934528_CDS GTGCTGGCTCTTTTTCATATATTTTATTGTATTACTACATTGGCTTTGGGAAGAGCTAAG 540

JW061356_CDS GTGCTGGCTCTTTTTCATATATTTTATTGTATTACTACATTGGCTTTGGGAAGAGCTAAG 540

*** **** ** *** * ** ** ***** * ** ** ******** ** ***

JN896629_CDS ATGAGAAAATGGAGGGCCTGGGAGGATGAAACAAAAACAATTGAGTACCAATTCTATAAC 486

JW054099_CDS ATGAGAAAATGGAGGGCCTGGGAGGATGAAACAAAAACAATTGAGTACCAATTCTATAAC 486

AY934528_CDS ATGAGTAGTTGGAAGGCATGGGAAAACGAAACAAGAACAGCTGAGTACCAATTTACAAAT 600

JW061356_CDS ATGAGTAGTTGGAAGGCATGGGAAAACGAAACAAGAACAGCTGAGTACCAATTTACAAAT 600

***** * **** *** ***** * ******* **** ************ **

JN896629_CDS GACCCTGAGAGGTTTAGGTTTGCAAGGGAGACCTCATTTGGACGTAGGCATATGCATTTT 546

JW054099_CDS GATCCTGAGAGGTTTAGGTTTGCAAGGGAGACCTCATTTGGACGTAGGCATATGCATTTT 546

AY934528_CDS GATCCAGAGAGATTTCGATTTGCTAGAGACACATCATTTGGAAGAAGACATTTGAGCTTT 660

JW061356_CDS GATCCAGAGAGATTTCGATTTGCTAGAGACACATCATTTGGAAGAAGACATTTGAGCTTT 660

** ** ***** *** * ***** ** ** ** ********* * ** *** ** ***

JN896629_CDS TGGAGCAAGTCGCCGGTGATGCTCTGGATAGTTTGTTTCTTCAGGCAATTCTTTTCATCA 606

JW054099_CDS TGGAGCAAGTCGCCGGTGATGCTCTGGATAGTTTGTTTCTTCAGGCAATTCTTTTCATCA 606

AY934528_CDS TGGACAAAAAATTCAGTGCTTCTATGGATTGTTTGTTTTTTCAGACAATTTGTAAGATCT 720

JW061356_CDS TGGACAAAAAATTCAGTGCTTCTATGGATTGTTTGTTTTTTCAGACAATTTGTAAGATCT 720

**** ** * *** * ** ***** ******** ***** ***** * ***

JN896629_CDS GTAGCAAAAGTTGACTATTTAACCCTTAGACATGGGTTCATGATGGCACATTTAACACCA 666

JW054099_CDS GTAGCAAAAGTTGACTATTTAACCCTTAGACATGGGTTCATGATGGCACATTTAACACCA 666

AY934528_CDS GTTCCAAAAGTTGATTATTTGACCCTACGTCATGGTTTTATCATGGCACATTTGGCACCT 780

JW061356_CDS GTTCCAAAAGTTGATTATTTGACCCTACGTCATGGTTTTATCATGGCACATTTGGCACCT 780

** ********** ***** ***** * ***** ** ** *********** ****

JN896629_CDS CAGAATCAAGAGAACTTTGATTTTCAAATATACATTAATAGAGCAGTTGACAAAGATTTC 726

JW054099_CDS CAGAATCAAGAGAACTTTGATTTTCAAATATACATTAATAGAGCAGTTGACAAAGATTTC 726

AY934528_CDS CAGAGCCAAATAAATTTTGATTTCCAAAAATATATTAAGAGGTCATTAGAAGAAGATTTC 840

JW061356_CDS CAGAGCCAAATAAATTTTGATTTCCAAAAATATATTAAGAGGTCATTAGAAGAAGATTTC 840

**** *** ** ******** **** *** ***** ** ** * ** ********

JN896629_CDS AAAGTTGTCGTGGGAATAAGTCCAGCATTATGGCTCTTCACGGTATTATATTTTCTATCC 786

JW054099_CDS AAAGTTGTCGTGGGAATAAGTCCAGCATTATGGCTCTTCACGGTATTATATTTTCTATCC 786

AY934528_CDS AAAGTAGTAGTTAGCATAAGTCCTCCAATTTGGTTCCTTGCTGTATTATTCCTACTCTTC 900

JW061356_CDS AAAGTAGTAGTTAGCATAAGTCCTCCAATTTGGTTCCTTGCTGTATTATTCCTACTCTTC 900

***** ** ** * ******** ** * *** ** * * ******* * ** * *

JN896629_CDS ACCACCGATGGAGTTTACTCGTATCTTTGGGTTCCATTTGTCCCACTCATTATAATATTG 846

JW054099_CDS ACCACCGATGGAGTTTACTCGTATCTTTGGGTTCCATTTGTCCCACTCATTATAATATTG 846

AY934528_CDS AATACTCATGGCTGGTATTCTTATCTGTGGCTACCATTCATTCCACTATTTGTGATATTG 960

JW061356_CDS AATACTCATGGCTGGTATTCTTATCTGTGGCTACCATTCATTCCACTATTTGTGATATTG 960

* ** **** ** ** ***** *** * ***** * ***** ** * ******

JN896629_CDS TTGGTTGGGACAAAACTTCAAATGATCATAACAGAAATGGGGGTTAGAATTTCAGAAAGG 906

JW054099_CDS TTGGTTGGGACAAAACTTCAAATGATCATAACAGAAATGGGGGTTAGAATTTCAGAAAGG 906

AY934528_CDS TTAGTAGGGACCAAATTACAAGTGATTATAACAAAAATGGGATTAAGAATTCAAGAAAGG 1020

JW061356_CDS TTAGTAGGGACCAAATTACAAGTGATTATAACAAAAATGGGATTAAGAATTCAAGAAAGG 1020

** ** ***** *** * *** **** ****** ******* * ****** *******

JN896629_CDS GGAGACATAGTGAAAGGTGTACCAGTGGTGGAGATCGGTGACCATCTTTTCTGGTTTAAT 966

JW054099_CDS GGAGACATAGTGAAAGGTGTACCAGTGGTGGAGATCGGTGACCATCTTTTCTGGTTTAAT 966

AY934528_CDS GGAGAAGTAGTGAAAGGTGTACCTGTGGTTCAGCCTGGAGATGATTTATTTTGGTTTAAT 1080

JW061356_CDS GGAGAAGTAGTGAAAGGTGTACCTGTGGTTCAGCCTGGAGATGATTTATTTTGGTTTAAT 1080

***** **************** ***** ** ** ** ** * ** *********

JN896629_CDS CGCCCTGGCCTTGTGCTTTTCTTCATTAACTTTGTCCTCTTTCAGAATGCGTTTCAAGTT 1026

JW054099_CDS CGCCCTGGCCTTGTGCTTTTCTTCATTAACTTTGTCCTCTTTCAGAATGCGTTTCAAGTT 1026

AY934528_CDS CGTCCTCGTCTTCTTCTTTATCTAATTAATTTTGTGCTTTTTCAGAATGCTTTTCAATTG 1140

JW061356_CDS CGTCCTCGTCTTCTTCTTTATCTAATTAATTTTGTGCTTTTTCAGAATGCTTTTCAATTG 1140

** *** * *** * **** * ***** ***** ** *********** ****** *

JN896629_CDS GCTTTCTTTGTTTGGAGTTGGTGGAAATTTGGTTTTCCATCCTGCTTTCATAGAAATGCT 1086

JW054099_CDS GCTTTCTTTGTTTGGAGTTGGTGGAAATTTGGTTTTCCATCCTGCTTTCATAGAAATGCT 1086

AY934528_CDS GCTTTCTTTGCTTGGACTTGGTATGAATTTGGGCTGAAATCTTGTTTCCATGACAAAACT 1200

JW061356_CDS GCTTTCTTTGCTTGGACTTGGTATGAATTTGGGCTGAAATCTTGTTTCCATGACAAAACT 1200

********** ***** ***** ******* * *** ** ** *** ** **

JN896629_CDS GCAGACCTAGCCATTAGGCTAACCATGGGAGTAATCATACAAGTCCATTGCAGCTATGTA 1146

JW054099_CDS GCAGACCTAGCCATTAGGCTAACCATGGGAGTAATCATACAAGTCCATTGCAGCTATGTA 1146

AY934528_CDS GAGGATATCGTCATTAGAATGACAATGGGGGTTCTTATTCAAATTCTTTGCAGCTATGTA 1260

JW061356_CDS GAGGATATCGTCATTAGAATGACAATGGGGGTTCTTATTCAAATTCTTTGCAGCTATGTA 1260

* ** * * ****** * ** ***** ** * ** *** * * *************

JN896629_CDS ACTCTCCCTCTATATGCCTTAGTTACTCAGATGGGTTCATCAATGAAGCCTATCATCTTT 1206

JW054099_CDS ACTCTCCCTCTATATGCCTTAGTTACTCAGATGGGTTCATCAATGAAGCCTATCATCTTT 1206

AY934528_CDS ACTCTTCCATTATATGCCCTTGTGACACAGATGGGATCATCAATGAAACCAACAATTTTC 1320

JW061356_CDS ACTCTTCCATTATATGCCCTTGTGACACAGATGGGATCATCAATGAAACCAACAATTTTC 1320

***** ** ******** * ** ** ******** *********** ** * ** **

JN896629_CDS GGTGATAATGTGGCAACAGCTCTTAGAAGCTGGCACAATACAGCGAAAAAGCGGGT--GA 1264

JW054099_CDS GGTGATAATGTGGCAACAGCTCTTAGAAGCTGGCACAATACAGCGAAAAAGCGGGT--GA 1264

AY934528_CDS AATGAAAGAGTAGCAACAGCATTGAGGAAGTGGCATCATGGTGCCAAGAAGCACATCAAA 1380

JW061356_CDS AATGAAAGAGTAGCAACAGCATTGAGGAAGTGGCATCATGGTGCCAAGAAGCACATCAAA 1380

*** * ** ******** * ** * ***** ** ** ** **** * *

JN896629_CDS GACATGGGCGGGTATCAG--AAAAC--ACCACTCCGATATCTAGCAGACCGGCCACACCA 1320

JW054099_CDS GACATGGGCGGGTATCAG--AAAAC--ACCACTCCGATATCTAGCAGACCGGCCACACCA 1320

AY934528_CDS GAGATCAACAAACATCATTCAAATCCAGCAACACCAATGTCAAGTAGGCCAACAACGCCC 1440

JW061356_CDS GAGATCAACAAACATCATTCAAATCCAGCAACACCAATGTCAAGTAGGCCAACAACGCCC 1440

** ** * **** *** * * ** ** ** ** ** ** ** * ** **

JN896629_CDS TTGCGTGGTACCTCCCCAGTTCACTTGCTACGTGGCTACCCAAAATATAACGAGGACAAT 1380

JW054099_CDS TTGCGTGGTACCTCCCCAGTTCACTTGCTACGTGGCTACCCAAAATATAACGAGGACAAT 1380

AY934528_CDS ACTCATGGCATGTCACCTGTCCATCTCCTACGCGGG-ATCCGGA------CGAG--TGAC 1491

JW061356_CDS ACTCATGGCATGTCACCTGTCCATCTCCTACGCGGG-ATCCGGA------CGAG--TGAC 1491

* *** * ** ** ** ** * ***** ** * ** * **** *

JN896629_CDS GTTCAAGCATATCCTCGAACATCGAATGTAGAAAATGAAG-GCTGGGCTAATGAAA---- 1435

JW054099_CDS GTTCAAGCATATCCTCGAACATCGAATGTAGAAAATGAAG-GCTGGGCTAATGAAA---- 1435

AY934528_CDS ATGGATGTGAGTCCACAAAGATCGAAT-TATAATGTGGACCATTGGGACATCGAGGGGTC 1550

JW061356_CDS ATGGATGTGAGTCCACAAAGATTGAAT-TATAATGTGGACCATTGGGACATCGAGGGGTC 1550

* * * *** * ** ** **** ** ** ** * **** * **

JN896629_CDS --CATCC-------------ACTGAG-----AATAAAGAT-------------------- 1455

JW054099_CDS --CATCC-------------ACTGAG-----AATAAAGAT-------------------- 1455

AY934528_CDS GTCATCTCCCACCCGATTCTACCAAGGTGGTGGTGGAGATGGCTCGTCTTCGCCGTCCCA 1610

JW061356_CDS GTCATCTCCCACCCGATTCTACCAAGGTGGTAGTGGAGATGGCTCGTCTTCGCCGTTCCA 1610

**** ** ** * ****

JN896629_CDS ----CATCAGG------AGGAGGGACAAATCCTGCAGCATGCC---TC-------CACT- 1494

JW054099_CDS ----CATCAGG------AGGAGGGACAAATCCTGCAGCATGCC---TC-------CACT- 1494

AY934528_CDS TATGCATCAAATTATTCAAAGTGGTCATGACTTACGTCATGACGACTCAGAAGGTCACGA 1670

JW061356_CDS TATGCATCAAACTATTCAAAGTGGTCATGACTTACATCATGACGACTCAGAAGGTCACGA 1670

***** * ** ** * * * **** * ** ***

JN896629_CDS -TCTA---TGCAACATCCGCATACTGATCAACATCAAATTGAGATTGCAATGTC----AG 1546

JW054099_CDS -TCTA---TGCAACATCCGCATACTGATCAACATCAAATTGAGATTGCAATGTC----AG 1546

AY934528_CDS GCCTAGTCTGCCACAAACGGCTCGTGACCAACACGAAGTCAACATTGCC-CGTCCAAGGG 1729

JW061356_CDS GCCTAGTCTGCCACAAACGGCTCTTGACCAACACGAAGTCAACATTGCC-CGTCCAAGGG 1729

*** *** *** ** * *** ***** ** * * ***** *** *

JN896629_CDS ATTTTACTTTTGG-----AAACAAATAG------- 1569

JW054099_CDS ATTTTACTTTTGG-----AAACAAATAG------- 1569

AY934528_CDS AATTCTCTTTTGATAAAAGAACAACTAGTGTATAA 1764

JW061356_CDS AATTCTCTTTTGATAAAAGAACAACTAGTGTATAA 1764

* ** ****** ***** ***
