# Supplementary figures and images for: Loss of Function in Mlo Orthologs Reduces Susceptibility of Pepper and Tomato to Powdery Mildew Disease Caused by Leveillula taurica
Source: PLoS One. 2013 Jul 29;8(7):e70723. doi: 10.1371/journal.pone.0070723 (PMC3726601; doi:10.1371/journal.pone.0070723)

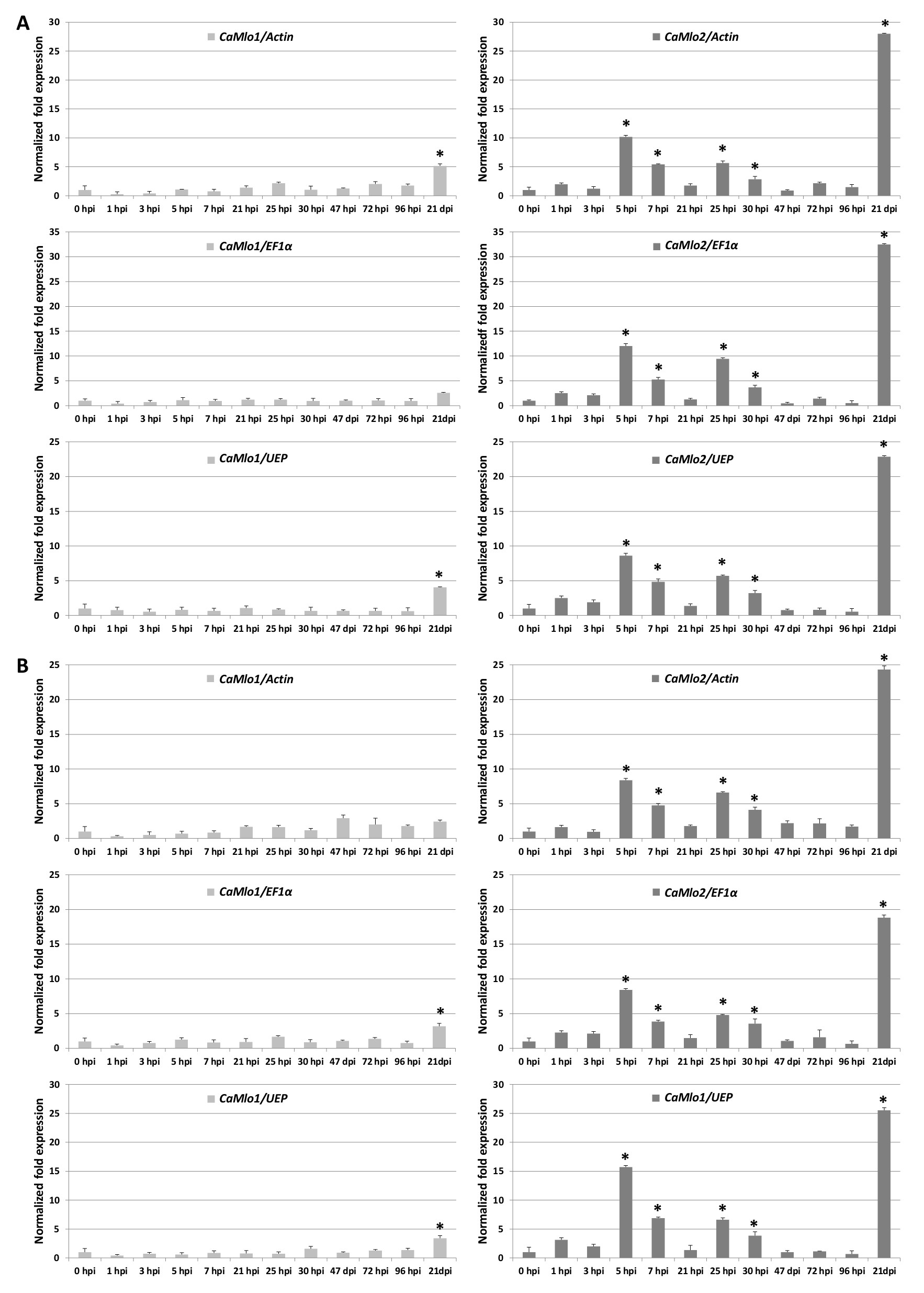

Supplement: Figure S2 — Expression profile of CaMlo genes measured by real time qRT-PCR in pepper leaves upon Leveillula taurica infection using three different reference genes. A, cultivar A. B, cultivar B. Columns indicate transcript fold changes with respect to non-inoculated plants (0 hours after inoculation (hpi)). Relative quantification was performed by using the ΔΔCt method and the reference genes CaActin, CaEF1α and CaUEP. Samples were taken from three whole pepper leaves per plant (the 3rd, 4th and 5th leaf) upon L. taurica infection at 0 hpi, 1 hpi, 3 hpi, 5 hpi, 7 hpi, 21 hpi, 25 hpi, 30 hpi, 47 hpi, 72 hpi, 96 hpi and 21 days post inoculation (dpi). Results are based on three individual pepper plants per time point. Bars refer to standard errors of the biological replicates and asterisks refer to significant differences with respect to non-inoculated plants, inferred by mean comparisons by Student's t-test. (TIF) [file pone.0070723.s002.tif]

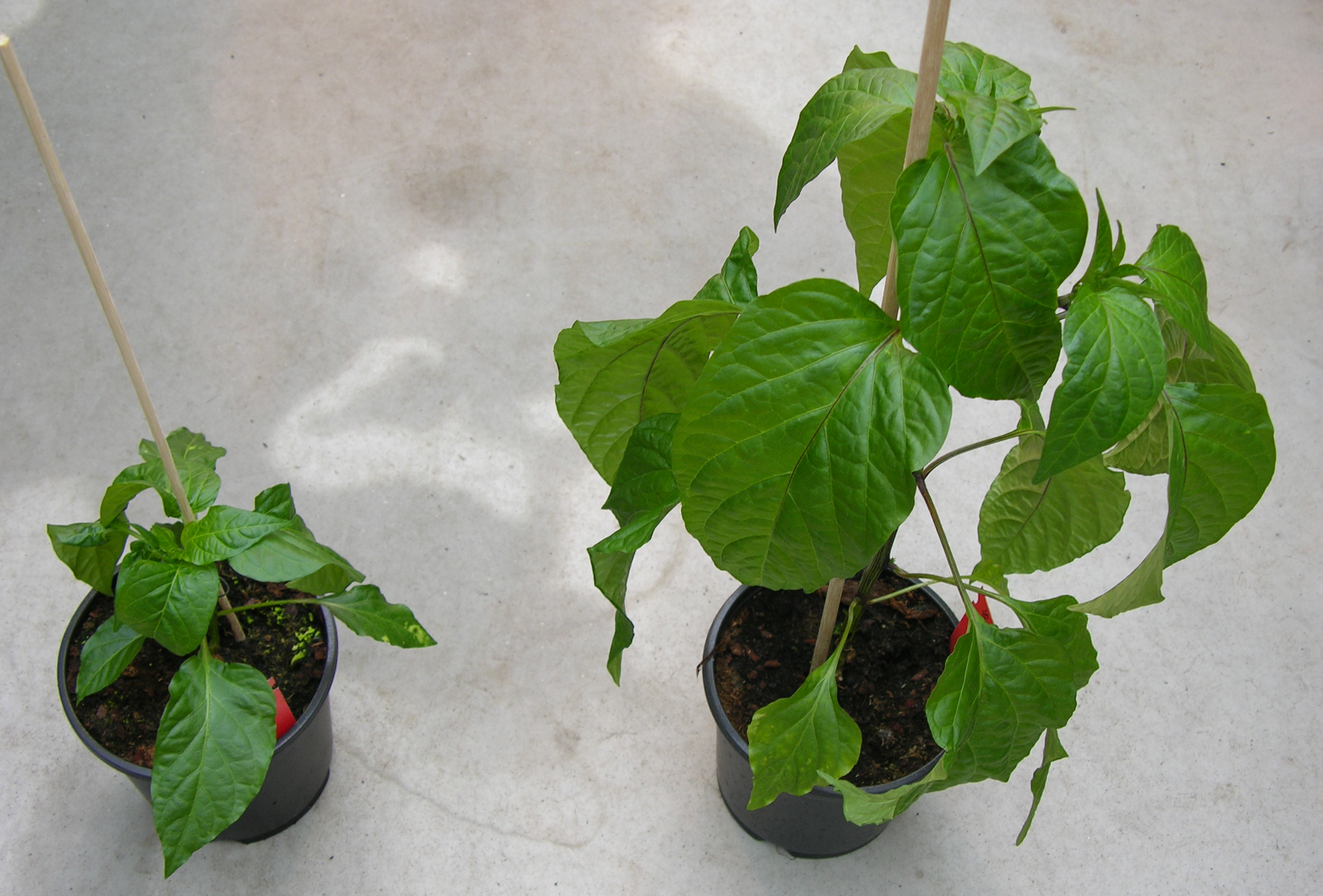

Supplement: Figure S3 — Eight out of ten plants in which the CaMlo2 gene is silenced with the VIGS construct CaMlo2-a (left) show an obvious decrease in size compared with plants in which CaMlo1 is silenced (right). (TIF) [file pone.0070723.s003.tif]
