# Supplementary material for: Loss of Function in Mlo Orthologs Reduces Susceptibility of Pepper and Tomato to Powdery Mildew Disease Caused by Leveillula taurica
Source: PLoS One. 2013 Jul 29;8(7):e70723. doi: 10.1371/journal.pone.0070723 (PMC3726601; doi:10.1371/journal.pone.0070723)
Supplement: Table S1 — Primers used for qRT-PCR gene expression analyses. (DOCX) [file pone.0070723.s005.docx]

# Table S1. Primers used for qRT-PCR gene expression analyses.

| **Gene** | **Target size** | **Forward primer sequence (5’→3’)** | **Reverse primer sequence (5’→3’)** | **Reference** |
| --- | --- | --- | --- | --- |
| *CaMlo1* | 235 bp | CAAACATCATTCAAATCCAGCAACACCA | AATTTGATGCATATGGGACGGCGAAGAC | This work |
| *CaMlo2* | 199 bp | CTGGCACAATACAGCGAAAA | TTCATTAGCCCAGCCTTCAT | This work |
| *CaActin* | 128 bp | ATCCCTCCACCTCTTCACTCTC | GCCTTAACCATTCCTGTTCCATTATC | [43] |
| *CaUBI-3* | 204 bp | TGTCCATCTGCTCTCTGTTG | CACCCCAAGCACAATAAGAC | [45] |
| *CaGAPDH* | 256 bp | ATGATGATGTGAAAGCAGCG | TTTCAACTGGTGGCTGCTAC | [45] |
| *CaEF1α* | 134 bp | TGAAGAATGGTGATGCTGGC | GACAACACCAACAGCAACAG | [46] |
| *CaUEP* | 210 bp | CCGACTACAACATCCAGAAG | CACACTCAGCATTAGGACAC | [46] |
