# Supplementary material for: Loss of Function in Mlo Orthologs Reduces Susceptibility of Pepper and Tomato to Powdery Mildew Disease Caused by Leveillula taurica
Source: PLoS One. 2013 Jul 29;8(7):e70723. doi: 10.1371/journal.pone.0070723 (PMC3726601; doi:10.1371/journal.pone.0070723)
Supplement: Table S2 — Primer pairs used to prepare VIGS constructs. (DOCX) [file pone.0070723.s006.docx]

# Table S2. Primer pairs used to prepare VIGS constructs.

| **Vector name** | **Target size** | **Forward primer sequence (5’→3’)** | **Reverse primer sequence (5’→3’)** |
| --- | --- | --- | --- |
| VIGS:CaMlo1-a | 493 bp* | CGGTTGCCGTAGTTTGTTTT | TGGAATGAATGGTAGCCACA |
| VIGS:CaMlo1-b | 667 bp** | TGTGGCTACCATTCATTCCA | GGCTCGTGACCTTCTGAGTC |
| VIGS:CaMlo2-a | 202 bp | caccTGTCCCCAAAAGTGTTGGTT | TAACCTGCCCAAAGCAAAAG |
| VIGS:CaMlo2-b | 239 bp | caccCGTGGGAATAAGTCCAGCAT | CAGGGCGATTAAACCAGAAA |

 * The PCR fragment of *CaMlo1-a* should be 896 bp. Due to the fact that the amplified PCR product contains an *Eco*RI digestion site at 403 bp the target fragment in the pTRV2 vector of *VIGS:CaMlo1-a* is 493bp.

** The PCR fragment of *CaMlo1-b* should be 748 bp. Due to the fact that the amplified PCR product contains an *Eco*RI digestion site at 81 bp the target fragment in the pTRV2 vector of *VIGS:CaMlo1-b* is 667 bp.
